# Microbial Community Dynamics in Soil Depth Profiles Over 120,000 Years of Ecosystem Development

**DOI:** 10.3389/fmicb.2017.00874

**Published:** 2017-05-19

**Authors:** Stephanie Turner, Robert Mikutta, Sandra Meyer-Stüve, Georg Guggenberger, Frank Schaarschmidt, Cassandre S. Lazar, Reiner Dohrmann, Axel Schippers

**Affiliations:** ^1^Geomicrobiology, Federal Institute for Geosciences and Natural ResourcesHanover, Germany; ^2^Soil Science and Soil Protection, Martin Luther University Halle-WittenbergHalle, Germany; ^3^Institute of Soil Science, Leibniz Universität HannoverHanover, Germany; ^4^Institute of Biostatistics, Leibniz Universität HannoverHanover, Germany; ^5^Aquatic Geomicrobiology, Institute of Ecology, Friedrich Schiller University JenaJena, Germany; ^6^Technical Mineralogy and Clay Mineralogy, Federal Institute for Geosciences and Natural ResourcesHanover, Germany

**Keywords:** *Archaea*, *Bacteria*, *Bathyarchaeota*, chronosequence, soil depth, subsoil, pyrosequencing, qPCR

## Abstract

Along a long-term ecosystem development gradient, soil nutrient contents and mineralogical properties change, therefore probably altering soil microbial communities. However, knowledge about the dynamics of soil microbial communities during long-term ecosystem development including progressive and retrogressive stages is limited, especially in mineral soils. Therefore, microbial abundances (quantitative PCR) and community composition (pyrosequencing) as well as their controlling soil properties were investigated in soil depth profiles along the 120,000 years old Franz Josef chronosequence (New Zealand). Additionally, in a microcosm incubation experiment the effects of particular soil properties, i.e., soil age, soil organic matter fraction (mineral-associated vs. particulate), O_2_ status, and carbon and phosphorus additions, on microbial abundances (quantitative PCR) and community patterns (T-RFLP) were analyzed. The archaeal to bacterial abundance ratio not only increased with soil depth but also with soil age along the chronosequence, coinciding with mineralogical changes and increasing phosphorus limitation. Results of the incubation experiment indicated that archaeal abundances were less impacted by the tested soil parameters compared to *Bacteria* suggesting that *Archaea* may better cope with mineral-induced substrate restrictions in subsoils and older soils. Instead, archaeal communities showed a soil age-related compositional shift with the *Bathyarchaeota*, that were frequently detected in nutrient-poor, low-energy environments, being dominant at the oldest site. However, bacterial communities remained stable with ongoing soil development. In contrast to the abundances, the archaeal compositional shift was associated with the mineralogical gradient. Our study revealed, that archaeal and bacterial communities in whole soil profiles are differently affected by long-term soil development with archaeal communities probably being better adapted to subsoil conditions, especially in nutrient-depleted old soils.

## Introduction

Soil microbial communities mediate key processes in soil ecosystem functioning including organic matter (OM) degradation, nutrient cycling, and mineral weathering. During the early stage of pedogenesis heterotrophic and phototrophic pioneer microorganisms are responsible for biological weathering of the bedrock material and create interfaces for OM and nutrient turnover, e.g., in biofilms (Tscherko et al., 2003; Nemergut et al., 2007; Schulz et al., 2013; Haynes, 2014). Thereby they provide favorable conditions for the colonization by plants (Schulz et al., 2013). With ongoing soil development and ecosystem progression, an increasing plant biomass causes an accumulation of OM that is accompanied by an increase in microbial cell numbers, biomass and activity of heterotrophic microorganisms (Richardson et al., 2004; Brankatschk et al., 2011; Turner et al., 2014). The archaeal community composition showed a shift during the first 110-years of soil development at a receding Swiss glacier (Zumsteg et al., 2012) and Nicol et al. (2005) reported changes within the phylum *Crenarchaeota* during 9500 years of succession for an Austrian glacier foreland. Similarly, many studies reported that the bacterial community composition considerably changed during progression with highest bacterial species turnover rates during the first years (Nemergut et al., 2007; Schütte et al., 2009; Wu et al., 2012; Zumsteg et al., 2012; Jangid et al., 2013a,b).

A soil chronosequence, i.e., soils of different ages that derived from the same parent material under similar climatic conditions, provides the unique opportunity for investigating microbial patterns with regard to soil development (Stevens and Walker, 1970). While microbial function and community composition dynamics during the development of young to intermediate-aged soils are already well investigated (Tscherko et al., 2003; Brankatschk et al., 2011; Zumsteg et al., 2012; Schulz et al., 2013), the knowledge about long-term dynamics is limited. There are a few studies analyzing bacterial communities of topsoils over several thousand years of ecosystem development including not only progressive but also retrogressive stages. Retrogression occurs after thousands to millions years when the ecosystem undergoes a decline in nutrient availability, productivity, and plant biomass (Peltzer et al., 2010). The diversity of bacterial communities decreased during retrogression coinciding with a depletion of soil phosphorus (P) (Jangid et al., 2013a,b; Uroz et al., 2014). However, there is a lack of information about archaeal community composition dynamics during retrogression.

Soil chronosequences are also an excellent tool to identify the environmental parameters that shape the microbial community composition during soil development. Most studies found distinct bacterial communities along the soil development gradient that were linked to changes in soil pH, carbon (C), and nutrient concentrations such as nitrogen (N) and P, or the C:N ratio (Zumsteg et al., 2012; Jangid et al., 2013a; Uroz et al., 2014; Freedman and Zak, 2015). In contrast, archaeal communities seem to be related to plant cover and N content (Zumsteg et al., 2012).

While most of these studies focus on topsoil communities it is still poorly understood how subsoil communities develop with ongoing soil age and which parameters are important in shaping these communities. Subsoils considerably differ in environmental conditions compared to topsoils, e.g., the concentrations of C and nutrients steeply decrease with soil depth (Hansel et al., 2008; Turner et al., 2014; Stone et al., 2015). Accordingly, subsoils harbor distinct microbial communities adapted to these energy and substrate limited conditions (Blume et al., 2002; Fierer et al., 2003; Hansel et al., 2008; Hartmann et al., 2009). Furthermore, the content of iron (Fe) and aluminum (Al) (hydr)oxides and clay minerals increase not only with increasing soil depth, but also most notably with increasing soil age (Tarlera et al., 2008; Mikutta et al., 2009; Turner et al., 2014). Tarlera et al. (2008) investigated bacterial communities of subsoil B horizons along a 77,000-years dune chronosequence and found a strong relationship between community structure and soil age, but did not further analyze the relationship to specific soil properties. Sorption of OM and nutrients such as P to reactive minerals may restrict substrate availability, particularly in subsoil environments, thus intensifying substrate limitation and potentially facilitate microbial communities adapted to these conditions. Further, the presence of particulate OM entering the topsoil as aboveground litter or roots may also induce differences in microbial community composition in comparison to subsoil, which are dominated by OM associated with minerals (Mikutta et al., 2009; Kleber et al., 2015).

Therefore, the main research questions of this study were (i) how microbial abundances and community composition develop in whole soil profiles along a long-term soil development gradient, and (ii) how microbial communities are shaped by soil properties during soil development with special consideration of microbial communities in mineral soils. To address these questions we investigated microbial abundances via quantitative PCR (qPCR) and community patterns via pyrosequencing of archaeal and bacterial 16S rRNA genes in soils along the 120,000 (120 kyr) years old Franz Josef chronosequence, New Zealand. The Franz Josef chronosequence gives the valuable opportunity to study patterns of long-term ecosystem development and is already well investigated in terms of vegetation and topsoil bacterial communities (Richardson et al., 2004; Jangid et al., 2013b), thus, providing background information for data interpretation. In addition, this soil chronosequence formed a C and nutrient gradient with highest C and N contents at the intermediate-aged sites and a sharp decline in total P content with ongoing soil development (Richardson et al., 2004; Turner et al., 2014). Further, the chronosequence is characterized by a mineralogical gradient that is characterized by an increase in Fe and Al (hydr)oxides and clay-sized minerals with soil age (Turner et al., 2014). High precipitation creates regularly water-saturated conditions in the soils of the chronosequence resulting in low oxygen availability and changes in the redox regime. Due to the complexity of environmental gradients (C, nutrients and soil mineralogy) along the Franz Josef chronosequence we additionally conducted a microcosm incubation experiment to disentangle the effects of the different soil properties on microbial communities. Therefore, we investigated microbial abundances via qPCR and microbial community patterns via T-RFLP and compared these between the different experimental incubation treatments, i.e., soil age, soil OM fraction (mineral-associated vs. particulate), O_2_ status, and C and P additions, covering the most relevant soil parameters that changes along the soil chronosequence.

## Materials and Methods

### Site Description and Soil Sampling

Soils of the Franz Josef chronosequence (∼43°S, 170°E) have developed due to glacier advance and retreat from greywacke and mica schist and are located on the West Coast of the South Island of New Zealand (Almond et al., 2001). The area is characterized by humid temperate climate with high mean annual precipitation (3500–6500 mm) and is covered by rainforest (Richardson et al., 2004).

**FIGURE 1:**
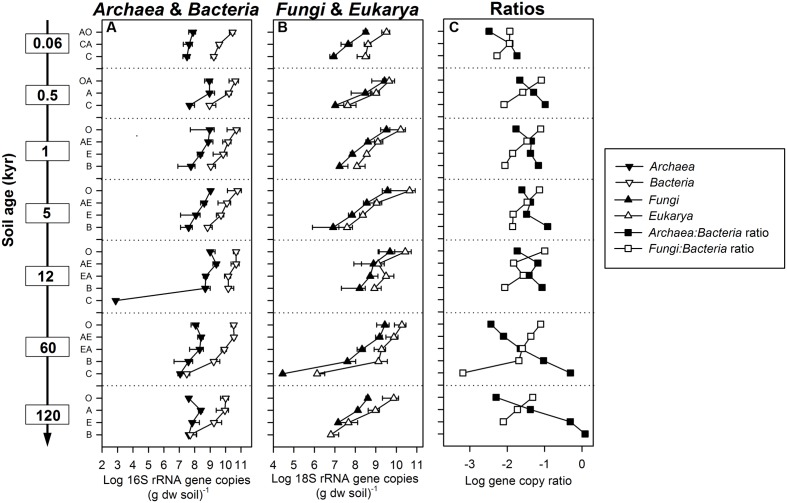
**Abundances of SSU rRNA gene copies in different soil horizons (O, OA, A, AO, AE, E, EA, B, C, CA) from depth profiles of 0.06, 0.5, 1, 5, 12, 60, and 120 kyr old soils along the Franz Josef chronosequence.** **(A)** *Archaea* and *Bacteria* 16S (assay Nadkarni et al., 2002), and **(B)** *Fungi* and *Eukarya* 18S rRNA gene copy numbers per gram soil dry weight (dw). Error bars indicate standard deviation of three parallel soil profiles. **(C)** Ratio of archaeal to bacterial, and fungal to bacterial SSU rRNA gene copy numbers.

Seven sites ranging from 0.06 to 120 kyr were sampled in January 2012 (Supplementary Figure S1; Stevens, 1968; Almond et al., 2001). At each site, three soil profiles (replicates) up to one meter depth were excavated and each horizon was sampled. Details of site characteristics and soil properties are given in Turner et al. (2014). Soil profiles contain organic topsoil (O horizon), mineral topsoil (A horizon), and subsoils with an eluvial horizon (E horizon), mineral subsoil (B horizon), and the parent material for soil genesis (C horizon); the respective horizons per site are shown in **Figure 1**. For molecular analyses, soil samples were taken with a sterile lab spoon, frozen and stored at -20°C until analysis. For the microcosm incubation experiment, A horizons of four selected sites (0.5, 5, 12, and 120 kyr) were sampled in February 2014. Soil samples were kept at <8°C prior to incubation.

### Nucleic Acid Extraction and Quantification of Small Subunit (SSU) rRNA Genes

Nucleic acids of all soil samples were extracted in triplicate (or in duplicate for the microcosm incubation experiment) using the FastDNA^®^Spin Kit for Soil (MP Biomedicals, Santa Ana, CA, United States) with modifications according to Webster et al. (2003) and DNA extracts were pooled.

Quantification of small subunit (SSU) rRNA genes of *Archaea*, *Bacteria*, *Fungi*, and *Eukarya* were performed by qPCR for all soil samples derived from the sampling in 2012. For the qPCR a StepOnePlus^TM^ Real-Time PCR System (Applied Biosystems, Life Technologies, Carlsbad, CA, United States) was used with either TaqMan^®^(*Bacteria* according to Nadkarni et al., 2002 and *Eukarya*) or SYBR^®^Green I chemistry (all others). All qPCRs contained 5 μL mastermix, 1 μL BSA (3 g L^-1^; Sigma–Aldrich, St. Louis, MO, United States), primers (**Table 1**), 1 μL DNA template and filled to the final volume of 10 μL with dH_2_O. As mastermixes we used Platinum SYBR Green qPCR SuperMix-UDG with ROX (Life Technologies, Carlsbad, CA, United States) for *Archaea* and *Bacteria* (Gray et al., 2011), PerfeCTa qPCR ToughMix ROX (Quanta Biosciences, Gaithersburg, MD, United States) for *Bacteria* (Nadkarni et al., 2002) and *Eukarya*, and FastStart Universal SYBR Green Master (ROX) (Roche, Rotkreuz, Switzerland) for *Fungi*. Standards were made from purified PCR product obtained from whole genome extracted from pure cultures: *Methanosarcina barkeri* (*Archaea*), *Escherichia coli* (*Bacteria* – Nadkarni et al., 2002), *Pseudomonas stutzeri* (*Bacteria* – Gray et al., 2011), and *Fusarium oxysporum* (*Fungi*). For the *Eukarya* assay we used fish sperm DNA as standard. For SYBR^®^Green I assays the product specificity was confirmed by melt curve analysis and the product size was verified by agarose gel electrophoresis. Template DNA was used in three dilutions to check for inhibition by co-extracted PCR inhibitors. Standard DNA, template DNA, and non-template control were run in three replicates. Abundances were reported in SSU rRNA gene copy numbers per gram dry weight of soil. Gene copy numbers of the incubation experiment were normalized to initial soil fraction organic C (OC) content (Supplementary Table S1) to account for the variability of the OC content in initial soil samples allowing a comparison of incubation experiment derived differences.

**Table 1:** Primers and conditions for qPCR assays.

Primer	Sequences (5′ – 3′) & qPCR conditions	Primer concentration (μM)	Efficiency (%)	Reference
**Archaeal 16S rRNA gene**			
Arch915F	AGG AAT TGG CGG GGG AGC AC	0.4	94.2–96.9	Kubo et al., 2012
Arch1059R	GCC ATG CAC CWC CTC T			
	95°C-5 min;			
	40×: 95°C-15 s, 60°C-45 s;			
	95°C-45 s			
**Bacterial 16S rRNA gene**			
Bac340F	TCC TAC GGG AGG CAG CAG T	0.1	87.8–91.5	Nadkarni et al., 2002
Bac806R	GGA CTA CCA GGG TAT CTA ATC CTG TT			
Bac probe	FAM-CGT ATT ACC GCG GCT GCT GGC			
	AC-TAMRA			
	50°C-2 min, 95°C-10 min;			
	40×: 95°C-15 s, 60°C-1 min			
U1048F	GTG ITG CAI GGI IGT CGT CA	0.25	93.1–95.5	Gray et al., 2011
U1371	ACG TCI TCC ICI CCT TCC TC			
	95°C-7 min;			
	40×: 95°C-30 s, 60.5°C-30 s, 72°C-40 s;			
	95°C-45 s			
**Fungal SSU 18S rRNA gene**				
nu-SSU-0817-F	TTA GCA TGG AAT AAT RRA ATA GGA	0.5	94.5–97.8	Borneman and Hartin, 2000
nu-SSU-1196-R	TCT GGA CCT GGT GAG TTT CC			
	95°C-10 min;			
	40×: 95°C-1 min, 56°C-1 min, 72°C-1 min;			
	95°C-1 min			
**Eukaryotic 18S rRNA gene**			
VIC	Probe and primers by Applied Biosystems	0.05	90.3–93.9	Applied Biosystems, 2002
	50°C-2 min, 95°C-10 min;			
	40×: 95°C-15 s, 60°C-1 min			


### Tag-Encoded Pyrosequencing of Archaeal and Bacterial 16S rRNA Genes

Archaeal and bacterial 16S rRNA genes of selected soil samples (sampling in 2012; Supplementary Figure S2) were amplified with the tag-encoded primer pairs Arch344f/Arch915r (Stahl and Amann, 1991; Sørensen et al., 2004) for *Archaea* and Bac341f/Bac785r (Herlemann et al., 2011) for *Bacteria*. All PCRs were performed using a total volume of 50 μL containing 5 μL of 10× polymerase buffer, 10 μL dNTPs (each 1 mM; Fermentas, Thermo Fisher Scientific, Waltham, MA, United States), 3 μL bovine serum albumin (3 g L^-1^), 2.5 μL of each primer (10 μM), 0.5 μL 1.25 U polymerase (*Pfu* DNA Polymerase, Promega, Madison, WI, United States), 24.5 μL dH_2_O and 2 μL template DNA. Touch-down PCR was performed with the following conditions: 95°C for 1.5 min, 10 touch-down cycles of 95°C for 1 min, 62°C (*Archaea*) and 65°C (*Bacteria*) for 30 s (decreasing by 1°C in each cycle), 72°C for 2 min, and 25 cycles of 95°C for 1 min, 52°C (*Archaea*) and 55°C (*Bacteria*) for 30 s, 72°C for 2 min, followed by 72°C for 5 min. The PCR products were checked for correct fragment size using agarose gel electrophoresis, cleaned up with the QIAquick PCR Purification Kit (QIAGEN, Hilden, Germany), and pooled in equimolar amounts. Adaptor ligation and sequencing of archaeal and bacterial amplicons were conducted by GATC Biotech (Konstanz, Germany) with the GS FLX System (Roche, Basel, Switzerland).

Sequences were sorted according to primers and tags, denoised according to Quince et al. (2009) and trimmed with mothur 1.29 (Schloss et al., 2009). Briefly, sequences with ≥1 mismatch to primer or tag sequence, ≥1 ambiguity or ≥8 homopolymers were removed. Tags were removed and sequences were aligned with SINA 1.2.11 (Pruesse et al., 2012). Chimeric sequences were identified using the UCHIME algorithm (Edgar et al., 2011) implemented in the mothur program package and removed. Remaining sequences were clustered to operational taxonomic units (OTUs) at 97% sequence identity level and *Bacteria* were classified with SILVA database release 115 (Quast et al., 2013). Richness estimator (Chao1) and Shannon diversity index were calculated for archaeal and bacterial OTUs using mothur.

The phylogenetic 16S rRNA gene trees for the phyla *Bathyarchaeota*, *Woesearchaeota*, *Thaumarchaeota* and *Euryarchaeota* were calculated with ARB (Ludwig et al., 2004) using the neighbor-joining method based on Jukes–Cantor distances. Therefore, 16S rRNA gene sequences were aligned with SINA (Pruesse et al., 2012).

### Soil Microcosm Incubation Experiment

To evaluate the effect of specific soil characteristics (O_2_ status, soil fraction, soil age, C, and P addition) on microbial abundances and community composition we set up a microcosm incubation experiment with soils of the Franz Josef chronosequence (Supplementary Figure S3; Turner et al., 2017). Therefore, we used different soil fractions (bulk soil, mineral-associated OM, and particulate OM) of A horizons of four soil ages (0.5, 5, 12, and 120 kyr). For soil density fractionation, 25 g of soil were dispersed in 125 mL sodium polytungstate solution (ρ = 1.6 g cm^-3^; Cerli et al., 2012) and sonicated for 9 min 38 s with 60 J mL^-1^ with an ultrasonic device (LABSONIC^®^, Sartorius Stedim Biotech GmbH, Göttingen, Germany). The lighter, particulate OM fraction (light fraction, LF) was separated from the heavy mineral-associated OM fraction (heavy fraction, HF) by decantation after deposition for 1 h and centrifugation for 10 min at 3500 *g*. To minimize possible negative effects of the sodium polytungstate on microbial activities (Blackwood and Paul, 2003; Crow et al., 2007), both fractions were washed with deionized water until the electrical conductivity was less than 50 μS cm^-1^, and freeze dried resulting in approximately 95 wt% of HF and 2 wt% of LF. For initial OC and total N concentrations see Supplementary Table S1. Toxic effects caused by tungsten residues in the fractionated samples could be ruled out because of similar mineralization rates of bulk soil compared to density fractions (Gentsch et al., 2015).

For each microcosm, 10 g (bulk or HF) or 1 g (LF) dried soil fraction sample were filled up to 20 g with sterile quartz powder (<125 μm, Carl Roth GmbH + Co. KG, Karlsruhe, Germany) into 125 mL serum vials. To evaluate the influence of C and P additions we tested different treatments in bulk soil and the HF: without any addition (wo), with NaH_2_PO_4_ (P; 500 μg PO_4_-P g^-1^ soil fraction), cellulose (C; 40 mg cellulose-C g^-1^ soil fraction C) or the addition of both (CP). The C and P treatments were not tested for the LF because we focused on the comparison between bulk soil vs. the HF and further had only very little amounts of LF material. Controls consisted of 20 g quartz powder only.

To further countervail the possible negative effect of the fractionation procedure on microbial activity, we inoculated each incubation sample with 3 mL of a soil slurry with an active, indigenous microbial community. Soil slurries consisted of fresh, wet soil of the corresponding sampling site (0.5, 5, 12, and 120 kyr) suspended in sterile Hoagland solution (without a P or N source, soil weight to volume ratio = 1:10). Samples were then adjusted to 60% water-holding capacity with sterile distilled water and carefully homogenized with a spatula. Replicate samples were incubated under oxic and anoxic conditions to analyze the effect of O_2_ status. For oxic incubation, serum vials were closed with polyethylene wool to allow gas exchange. Anoxic vials were fitted with septa and flushed repeatedly with helium. All samples were pre-incubated at 15°C for 10 days for equilibration and afterward the cellulose was added and the main-incubation started. For maintaining high humidity in oxic vials, a tray of water was placed into the incubator and the water content was checked every few days and, if necessary, readjusted. The loss of water in anoxic vials was negligible during incubation. All combinations of the tested factors (O_2_ status, soil fraction, soil age, and C and P addition) were prepared in triplicate resulting together with the controls in 222 incubation samples. After 125 days of incubation, all vials were sampled destructively for end-point molecular analyses including qPCR (assays for *Archaea*, *Bacteria* and *Fungi* as described above) and terminal restriction fragment length polymorphism (T-RFLP). Samples were frozen and stored at -20°C until analysis.

### Community Profiling of Incubation Samples via T-RFLP

Archaeal and bacterial community composition of incubation samples were analyzed via T-RFLP rather than pyrosequencing because for the incubation experiment the focus was on community patterns and a lower microbial diversity was expected than in the original soil samples. For amplification, DNA extracts were purified and concentrated with PowerClean^®^Pro DNA Clean-Up Kit (MO BIO Laboratories, Carlsbad, CA, United States). Archaeal 16S rRNA genes were amplified with primer pair Cy5-labeled 20F (Massana et al., 1997) and 915R (Stahl and Amann, 1991) and bacterial 16S rRNA genes with primer pair Cy5-labeled 8F (Frank et al., 2007) and 907R (Muyzer et al., 1995). The 25 μL PCR reaction volume contained 5 μL OneTaq^®^Standard Reaction Buffer (New England Biolabs, Ipswich, MA, United States), 5 μL dNTPs (each 1 mM; Fermentas, Thermo Fisher Scientific, Waltham, MA, United States), 1.5 μL BSA (3 g L^-1^), 0.6 μL (*Archaea*) or 0.4 μL (*Bacteria*) of each primer (10 μM), 0.125 μL OneTaq^®^Hot Start DNA Polymerase (5 U μL^-1^), 1–2.5 μL sample DNA and was filled up with dH_2_O. The thermal cycling protocol was 95°C for 5 min, 30 cycles of 95°C for 30 s, 57°C (*Archaea*) or 52°C (*Bacteria*) for 30 s, 68°C for 1 min 10 s and the final extension at 68°C for 10 min on a Biometra TProfessional Thermocycler (Analytik Jena AG, Jena, D). The PCR products length was checked on an agarose gel and digested with HaeIII and RsaI for *Archaea* or HaeIII and HhaI for *Bacteria* (all restriction enzymes were purchased from Thermo Scientific, Carlsbad, MA, United States) for 2 h at 37°C. Restriction fragments (T-RFs) were analyzed on a capillary sequencer (Beckman Coulter, GenomeLab^TM^ GeXP, CEQ8000, Fullerton, CA, United States). The T-RFLP data was noise filtered and aligned via T-REX (Culman et al., 2009).

For data analysis, T-RFs with a fragment size <65 or >600 bp were discarded. Relative abundances of T-RFs were calculated based on peak areas and only T-RFs with a relative abundance of ≥3% in all samples were included in further analysis.

### Data Analysis and Statistics

Soil properties of the Franz Josef chronosequence were analyzed by Turner et al. (2014) on the same samples and were used for statistical analysis (Supplementary Table S2). Gene copy numbers of the chronosequence samples were determined for every soil horizon and for the figures mean values were calculated for each horizon cluster (O, A, E, B, and C; *n* = 28). For statistical analysis we used all individual data points (*n* = 101). To explore the relationship between soil properties and SSU rRNA gene copy numbers we calculated spearman rank order correlation coefficients with R (version 3.2.3; R Core Team, 2015).

The effects of soil age, horizon, age × horizon interaction and depth within a horizon on SSU rRNA gene copy numbers were analyzed as fixed effects in a linear mixed effects model (‘lmer’ function of the ‘lme4’ package; Bates et al., 2015). Therefore, SSU rRNA gene copy number data were log-transformed because they ranged across several orders of magnitude, were right-skewed and showed increasing variance with increasing means. The variance between individual profiles was included as random effect to account for repeated measures within the same profile. The inspection of initial model residuals revealed that variances differed between topsoil (O, A) and subsoil (E, B, C) horizon clusters despite log-transformation. Thus, separate models were fitted to topsoil and subsoil data subsets. The main effects and interaction were tested in ANOVA following the model fit, all pairwise comparisons (Tukey-Test) of soil age groups within horizon clusters were performed based on least square means, after centering depth at each age × horizon clusters’ mean depth (package ‘lsmeans,’ Lenth, 2015).

Multivariate statistics for pyrosequencing community data was performed with Canoco 5 (v5.02; ter Braak and Šmilauer, 2012) on relative proportions of archaeal groups and bacterial genera. To illustrate differences in microbial communities between the samples, we conducted a principal component analysis (PCA) and used the parameters soil age and soil depth as supplementary variables. To assess the conditional (unique) effect of soil age and soil depth on microbial community composition we used variation partitioning and Monte Carlo permutation test (99,999 permutations). Redundancy analysis (RDA) with interactive forward selection and Monte Carlo permutation test (99,999 permutations) was used to identify the effect of soil properties that explained a significant proportion of variation in the microbial community composition. All tests were considered as significant at *P* < 0.05.

For the incubation experiment data set (bulk vs. HF samples), the main effects of O_2_ status, soil fraction, soil age, P and C addition on SSU rRNA gene copy numbers were determined by a five-way ANOVA including their interactions with R. Therefore, the measured parameters and residuals were checked for normal-distribution and log-transformed if necessary. Since we got significant results for almost all five factors for several parameters, we used a random effect model including the five factors and their two-way interactions to estimate the variance components using the ‘lmer’ function of the ‘lme4’ package (Bates et al., 2015) for determining the relative importance of each factor. Because in this relatively simple model the O_2_ status appeared to be the most important factor for the variation in most microbial abundance patterns, we also used variance component estimation in a more complex random effect model including the remaining four factors and their four-way interactions for the oxic and anoxic data subset.

To explore how the tested factors affect the archaeal and bacterial community composition in the incubation experiment we conducted a non-metric multidimensional scaling (NMDS) with Bray–Curtis distance measure and two dimensions based on relative T-RF abundances in Canoco 5. To test for significant differences in community composition (bulk vs. HF samples) between the levels of each factor we used a multiresponse permutation procedure (MRPP) with Bray–Curtis distance and 99,999 permutations. The MRPP is implemented in the vegan package (Oksanen et al., 2015) in R and generates the chance-corrected within group agreement statistic A. Values of A close to 0 correspond to no systematic differences between the levels of the tested factor, whereas values close to 1 indicate that items within each factor level are nearly identical, i.e., the tested factors define clearly different groups with mostly homogeneous community composition within a group.

### Sequence Deposition

Archaeal and bacterial 16S rRNA gene raw reads were deposited in NCBI Sequence Read Archive (SRA) under the BioProject ID PRJNA299489.

## Results

### Abundances of Archaea, Bacteria, Fungi and Eukarya along the Soil Chronosequence

Archaeal, bacterial, fungal, and eukaryotic SSU rRNA gene copy numbers were determined by qPCR (**Figures 1A,B**). Overall, in O horizons the copy numbers of all four taxa peaked at the intermediate-aged sites (1–12 kyr; Supplementary Table S3) and decreased with soil depth at all sites. With regard to soil depth, the maximum of the archaeal gene copy number was not always in O horizons as for all other groups but at some sites in A horizons (0.5, 12, 60, and 120 kyr). In subsoil horizons all SSU gene copy numbers showed an age effect with significant lower abundances at the oldest site except for the *Archaea* (Supplementary Table S3). The sum of archaeal and bacterial 16S rRNA gene copy numbers was positively correlated to total cell counts determined by Turner et al. (2014) (*r* = 0.91, *P* < 0.001). Interestingly, the ratio of *Archaea* to *Bacteria* not only increased with soil depth but also significantly with soil age in subsoils (**Figure 1C** and Supplementary Table S3). In contrast, the fungal to bacterial abundance ratio decreased with soil depth and was lowest at the youngest and the oldest site throughout the soil profile. All SSU rRNA gene copy numbers were highly positively correlated with soil OC, organic N (ON), ammonium and organic P (OP) content and negatively with pH (**Table 2** and Supplementary Table S4). Correlations between gene abundances and soil parameters such as the total P and nitrate content, or mineralogical properties such as particle size and content of pedogenic Fe and Al phases were weaker (Supplementary Table S4).

**Table 2:** Spearman rank order correlation coefficients for SSU rRNA gene copy numbers g^-1^ dry weight soil and soil properties of the soil chronosequence.

	OC	ON	OP	pH
*Archaea*	0.76	0.77	0.55	-0.75
*Bacteria*	0.89	0.89	0.73	-0.73
*Fungi*	0.88	0.88	0.66	-0.79
*Eukarya*	0.86	0.85	0.62	-0.71


*All correlations were significant (*P* < 0.001).*

### Archaeal and Bacterial Community Composition along the Soil Chronosequence

**FIGURE 2:**
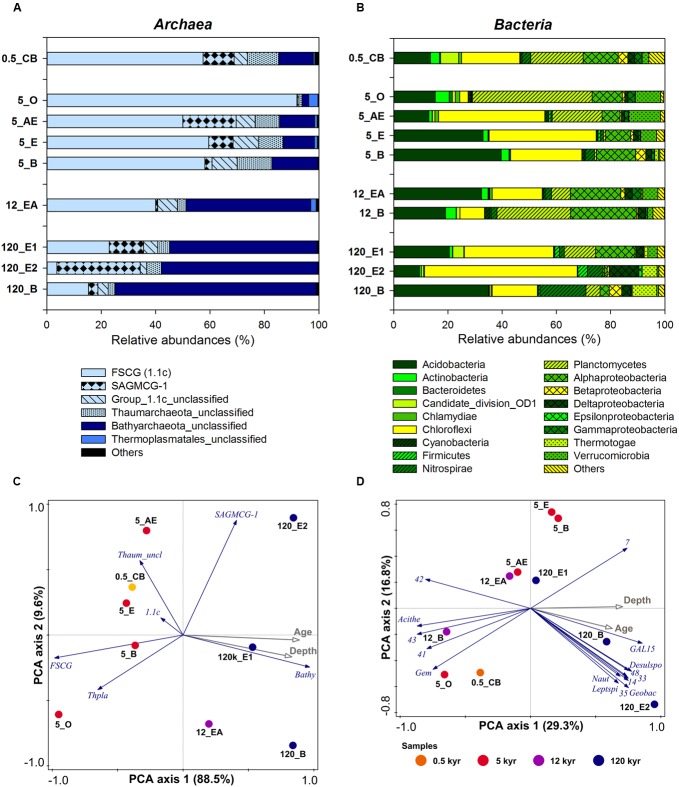
**Microbial community composition of selected original soil samples (0.5, 5, 12, and 120 kyr soil age) along the Franz Josef chronosequence based on pyrosequencing analysis.** Relative abundances (%) of **(A)** archaeal groups, and **(B)** bacterial phyla and classes (*Proteobacteria*). **(C)** Principal component analysis (PCA) of archaeal community composition based on relative abundances of archaeal groups with soil age and soil depth as supplementary variables (gray arrows). *1.1c*, *Thaumarchaeota 1.1c*; *Bathy*, *Bathyarchaeota*; *FSCG*, *Forest Soil Crenarchaeotal Group; SAGMCG-1*, *South African Gold Mine Crenarchaeotal Group 1*; *Thaum_uncl*, unclassified *Thaumarchaeota*, and *Thpla*, *Thermoplasmatales*. **(D)** PCA of bacterial community composition based on log-transformed relative abundances of bacterial genera (15 best-fitting as blue arrows) with soil age and soil depth as supplementary variables (gray arrows). *Acithe*, *Acidothermus*; *Desulspo*, *Desulfosporosinus*; *GAL15*, candidate division *GAL15;* Gem, *Gemmata*; *Geobac*, *Geobacter*; *Leptspi*, *Leptospirillum*; *Naut*, *Nautilia*. Uncultured genera belong to 7, *Acidobacteria*; 14, *Chloroflexi*; 33, 35, *Nitrospirae*; 41, *Planctomycetes*; 42, 43, *Alphaproteobacteria*; 48, *Deltaproteobacteria*. Symbol colors encode the different soil ages.

**FIGURE 3:**
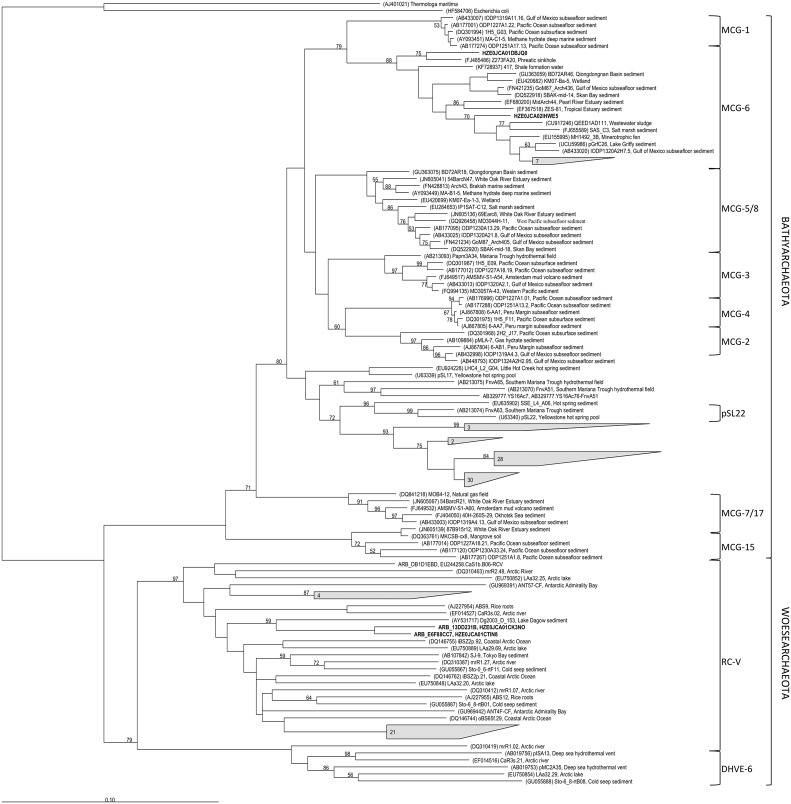
**Phylogenetic affiliation of *Bathyarchaeota* and *Woesearchaeota* 16S rRNA gene sequences from soils along the Franz Josef chronosequence (in bold or grouped as gray-shaded form including the number of the respective sequences).** Bootstrap values (in percent) are based on 1000 replicates (values below 50% are not shown). The scale bar indicates 10% estimated phylogenetic divergence.

The archaeal and bacterial community composition was analyzed using tag-encoded pyrosequencing of 16S rRNA genes. The pyrosequencing yielded a total of 592,752 reads, which were quality-filtered and then clustered to 2208 archaeal and 28,482 bacterial OTUs at 97% sequence identity level. The archaeal community composition was dominated by the *Forest Soil Crenarchaeotal Group (FSCG*, belong to *Group 1.1c*; Jurgens et al., 1997), the *South African Gold Mine Crenarchaeotal Group 1* (*SAGMCG-1*; Takai et al., 2001), both belonging to the *Thaumarchaeota* (Supplementary Figure S4), and the newly proposed phylum *Bathyarchaeota* (Meng et al., 2014), formerly known as *Miscellaneous Crenarchaeotal Group* (*MCG*) (**Figure 2A**). Interestingly, most sequences that belong to the *Bathyarchaeota* were affiliated with sequences of the pSL22 cluster, a sister group of the *MCG*, and only a few were closely related to the *MCG*, subgroup 6 (**Figure 3**). Furthermore, archaeal communities comprised members of the *Euryarchaeota* (e.g., *Thermoplasmatales*, *Rice Cluster I* and *Methanomicrobia*; Supplementary Figure S5) and *Woesearchaeota* (*Rice Cluster V*; **Figure 3**), however, both constituting only <1% of the total archaeal community. Archaeal diversity and richness decreased with increasing soil age (Supplementary Table S5).

There was a clear compositional shift with soil age characterized by a *FSCG* dominance at the young to intermediate-aged soils to a high abundance of the *Bathyarchaeota* at the older sites (**Figure 2C**). To examine the unique effect of soil age and soil depth on archaeal community composition we conducted variation partitioning and the results emphasized the stronger effect of soil age accounting for 17.8% of total adjusted variation compared to the effect of soil depth with 14.3% (both *P* < 0.05). The effect of soil properties on the archaeal community composition was analyzed by RDA using forward selection. The content of OP and pyrophosphate-extractable Fe (Fe_p_), representing Fe in metal-organic complexes, explained 75.8 and 9.5% of the total variation of archaeal community composition, respectively (both *P* < 0.05).

Compared to the *Archaea*, the bacterial community remained quite stable with ongoing soil development and the most abundant phyla across all samples were *Acidobacteria*, *Chloroflexi*, *Planctomycetes*, and *Proteobacteria* (**Figure 2B**). A PCA of the relative abundances of bacterial genera confirmed that samples did not cluster according to soil age (**Figure 2D**). This finding was confirmed by variation partitioning showing that the conditional effect of soil age and soil depth separately was not significant, but explained together (including their interaction) 10.3% of the bacterial community variation (*P* = 0.052). A RDA of relative proportions of bacterial genera with forward selection of soil properties showed that the content of nitrate and silt explained together a proportion of 39.2% of total adjusted variation (both *P* < 0.05). Instead of nitrate and silt content also pH (if picked first in the forward selection) can be a good predictor for community composition explaining 21.2% (*P* < 0.001) of the total variation. Similar to *Archaea*, bacterial diversity and richness also decreased with soil age (Supplementary Table S5).

### Incubation Experiment: Abundances of Archaea, Bacteria and Fungi

**FIGURE 4:**
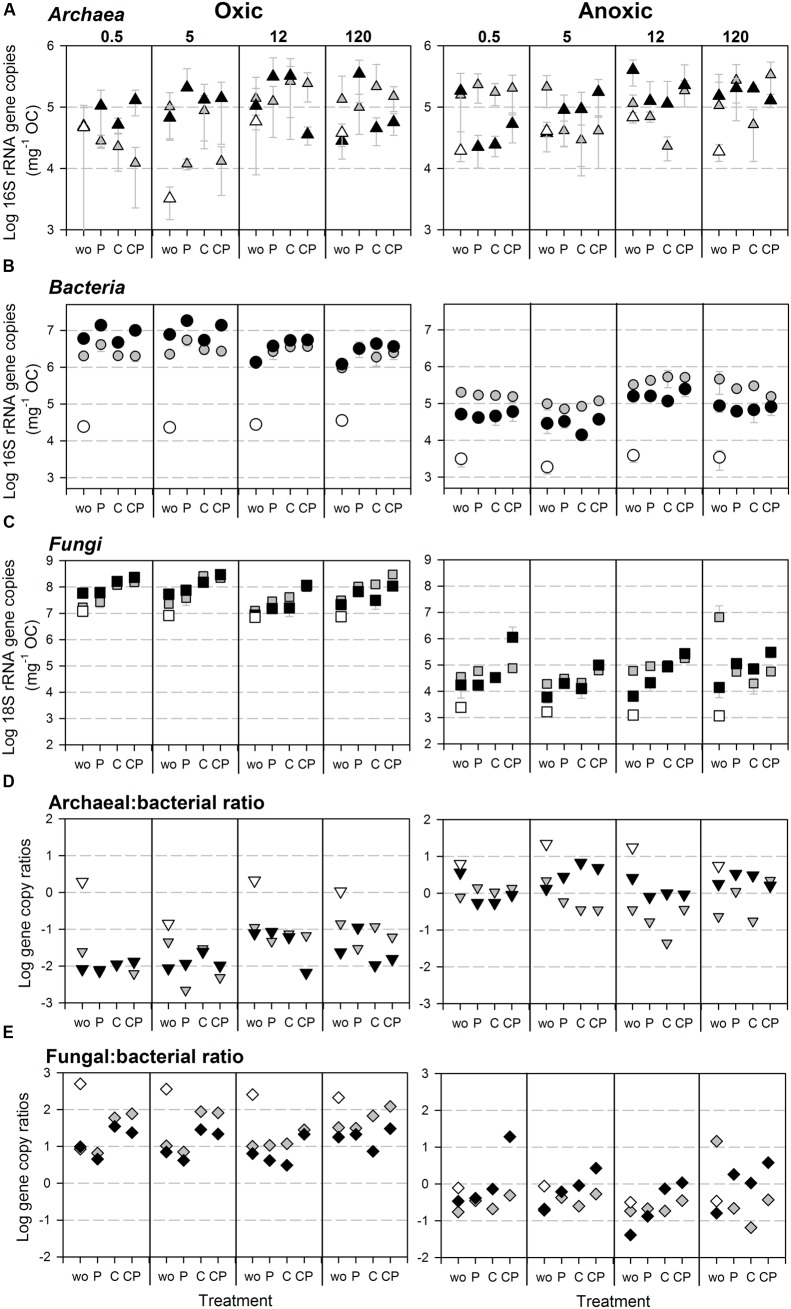
**Abundances of SSU rRNA gene copies normalized to initial OC content in the soil microcosm incubation experiment for** **(A)** *Archaea*, **(B)** *Bacteria* (assay Gray et al., 2011), and **(C)** *Fungi* as well as **(D)** archaeal to bacterial, and **(E)** fungal to bacterial SSU rRNA gene copy ratios under oxic (Left) and anoxic (Right) conditions. Gray shades of symbols indicate soil fraction: bulk (gray), HF (black), and LF (white, only wo-treatment). Every plot shows gene copy numbers of the differently aged soils (0.5, 5, 12, and 120 kyr), each with the four treatments (wo, P, C, and CP). Error bars indicate standard deviation of three parallel incubation samples.

**FIGURE 5:**
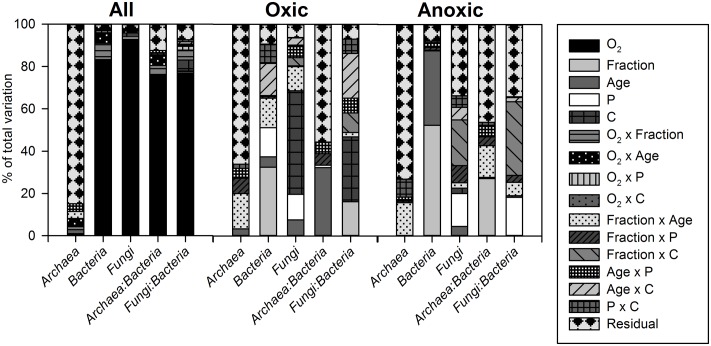
**Variance component estimation of factors (O_2_, fraction, soil age, P, and C addition and their two-way interactions) controlling microbial abundances and abundance ratios in the whole data set (all), and the oxic and anoxic subsets (without the LF, controls, and inoculum) for the soil microcosm incubation experiment**.

The variation in SSU rRNA gene copy numbers could be mainly explained by the O_2_ status for *Bacteria* and *Fungi*, but not for *Archaea* (**Figures 4A–C**, **5**). For bacterial 16S rRNA gene copy numbers the soil fraction was the second most important control with the effect depending on the O_2_ status (**Figure 5**): Under oxic conditions bacterial 16S rRNA gene copy numbers were higher for HF samples, whereas under anoxic conditions copy numbers were higher for bulk samples (**Figure 4B**). For the LF, all SSU rRNA gene copy numbers were similar or lower compared to bulk and HF samples with the greatest differences for *Bacteria* (**Figures 4A–C**). However, P and C addition did not significantly affect archaeal 16S rRNA gene copy numbers, whereas bacterial and fungal copy numbers mostly increased due to C and P addition in both bulk and HF samples, especially under oxic conditions (**Figures 4A–C** and Supplementary Tables S6, S7). The archaeal to bacterial abundance ratios showed higher values for HF samples under anoxic conditions, but were relatively similar under oxic conditions (**Figure 4D**). The C and CP treatment in HF samples resulted in higher fungal to bacterial abundance ratios (**Figure 4E**). The results of the variance component estimation with the more complex random effect model for the oxic and anoxic data subsets showed that despite the significance of three-way factor interactions (ANOVA), most variation in microbial abundance patterns could be explained by the effect of single factors or their two-way interactions (Supplementary Tables S6–S8).

### Incubation Experiment: Archaeal and Bacterial Community Composition

**FIGURE 6:**
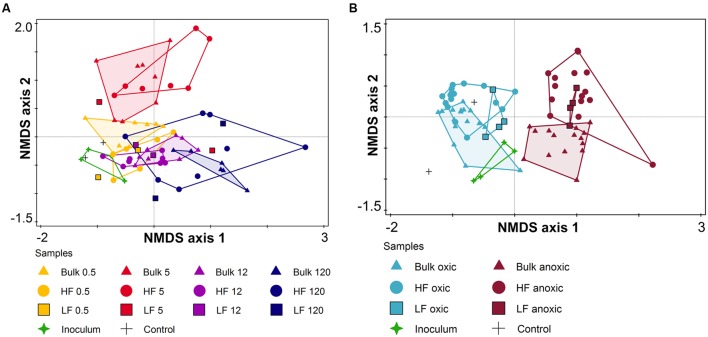
**Non-metric multidimensional scaling (NMDS) of** **(A)** archaeal (stress value = 0.218) and **(B)** bacterial community composition (stress value = 0.133) for the soil microcosm incubation experiment based on relative abundance of T-RFs (after digestion of PCR products with HaeIII) using Bray–Curtis distance measure. Different symbols encode the different soil fractions (triangle – bulk soil; circle – heavy fraction, HF; and square – light fraction, LF). Different colors encode the different soil ages (0.5, 5, 12, and 120 kyr) for archaeal community analysis **(A)** and O_2_ status (oxic and anoxic) for bacterial community analysis **(B)**.

Archaeal and bacterial community composition were analyzed via T-RFLP, each with two restriction enzymes resulting overall in 31 (HaeIII) and 23 T-RFs (RsaI) for *Archaea*, and 54 (HaeIII) and 46 T-RFs (HhaI) for *Bacteria*. Both were significantly affected by the soil fraction (bulk vs. HF; **Table 3**) and showed T-RFs that were unique for the respective fraction (Supplementary Table S9). Besides the soil fraction, the site age mainly affected the archaeal community composition followed by a slight effect of the O_2_ status (**Figure 6A**, **Table 3**, and Supplementary Figure S6A). However, archaeal communities of LF samples were only little affected by soil age and partially clustered to samples of a different soil age. On the contrary, the bacterial community composition showed distinct clusters for oxic and anoxic conditions (**Figure 6B**, **Table 3**, and Supplementary Figure S6B). Archaeal as well as bacterial community compositions were not significantly affected by the addition of P or C (**Table 3**).

**Table 3:** Effects of the different factors on microbial community composition in the soil microcosm incubation experiment (bulk and HF, without the LF) as revealed by multi-response permutation procedure (MRPP).

	A (O_2_)	A (Fraction)	A (Site age)	A (P)	A (C)
Archaea (HaeIII)	**0.047**	**0.026**	**0.220**	-0.008	-0.007
Archaea (RsaI)	**0.063**	**0.050**	**0.138**	-0.004	-0.011
Bacteria (HaeIII)	**0.178**	**0.048**	0.028	0.008	-0.007
Bacteria (HhaI)	**0.186**	**0.048**	**0.045**	0.004	-0.007


*A is the within-group agreement statistic, significant results at *P* < 0.01 are typed in bold.*

## Discussion

Our study provides the first insight into the dynamics of microbial abundances and communities in soil depth profiles over long-term ecosystem development including retrogression. Using the Franz Josef chronosequence gave us the unique opportunity to analyze community patterns of the soil microbiota linked to pedogenesis in an undisturbed soil environment. To explore the relevance of specific soil properties for microbial patterns during long-term ecosystem development, we set up a microcosm incubation experiment. While such experiments cannot entirely reflect the conditions in natural soil, they provide valuable insights into the interactions between individual soil characteristics and soil microbiota.

The soils along the Franz Josef chronosequence are covered by rainforest (Richardson et al., 2004) that provides, especially at the intermediate-aged sites (1–12 kyr), a large input of OM to topsoil horizons causing a high microbial biomass, high enzymatic activities (Turner et al., 2014), and a maximum of archaeal, bacterial, fungal, and eukaryotic SSU rRNA gene copy numbers in organic horizons at these sites (**Figures 1A,B** and Supplementary Table S3). This link between vegetation and belowground microbiota was supported by correlation analysis showing a strong relationship between soil OC, ON and OP contents, and microbial activities and abundances (microbial biomass, total cell counts, and SSU rRNA gene copy numbers) for whole soil profiles along the chronosequence (**Table 2**; Turner et al., 2014).

### Archaea rather than Bacteria Predominate Subsoils, Especially at Older Development Stages

The *Archaea* to *Bacteria* abundance ratio was lowest in the organic horizon and increased with soil depth pointing to a relative predominance of *Archaea* in subsoils (**Figure 1C**). Additionally, bacterial, fungal, and eukaryotic abundances were maximal in the organic horizon, whereas archaeal abundances were highest in mineral A horizons at some sites. These observations were in line with findings for a spruce forest, where a higher abundance of *Archaea* was determined via metagenomics for the mineral soil horizon compared to the organic horizon (Uroz et al., 2013). Archaeal 16S rRNA gene copy numbers in a soil profile of a boreal peatland even increased in the upper 50 cm and then remained almost stable to a depth of 175 cm (Lin et al., 2014). Accordingly, *Bacteria* and *Fungi* seem to dominate the OM-rich layer (O horizon) being responsible for the decomposition of particulate OM while the *Archaea* seem to be better adapted to subsoil conditions where OM generally becomes less available due to a multitude of soil environmental factors, including sorption to and stabilization by minerals (Schmidt et al., 2011; Kleber et al., 2015). Fertilization and manipulation studies have shown that archaeal to bacterial abundance ratios are negatively related to increasing soil C contents suggesting that soil *Archaea* might be rather oligotrophs (Fierer et al., 2007; Nemergut et al., 2010; Wessén et al., 2010; Chroňáková et al., 2015).

Interestingly, the *Archaea* to *Bacteria* abundance ratio not only increased with soil depth but also with soil age pointing to an increasing relative quantitative importance of *Archaea* in long-term soil development coinciding with a P-limitation and an increase in Fe and Al (hydr)oxides as well as other clay-sized minerals at older stages (Turner et al., 2014). However, our results of the incubation experiment showed that archaeal abundances were hardly affected by soil fraction (bulk vs. HF), soil age (including the mineralogical gradient) as well as C and P addition (**Figure 5** and Supplementary Table S6) showing that archaeal abundances were rather stable under the tested conditions. Based on an 18-months incubation experiment with artificial soils, Hemkemeyer et al. (2014) also reported only a weak influence of the clay mineral composition on archaeal abundances with no effect of the addition of the Fe oxide ferrihydrite. In addition, archaeal abundances increased slower than *Bacteria* or *Fungi* over the 18 months indicating slower growth rates and a better adaptation to nutrient-poor conditions. In general, the distribution across extreme habitats and a range of biochemical mechanisms to cope with extreme conditions suggest that the adaptation to chronic energetic stress is a crucial factor that ecologically and evolutionary distinguish *Archaea* from *Bacteria* (Valentine, 2007).

In contrast, bacterial abundances were significantly impacted by almost all tested factors in our incubation experiment (Supplementary Table S6) with the largest effects of the O_2_ status and the soil fraction (**Figure 5**). Similarly, Hemkemeyer et al. (2014) reported a pronounced effect of mineral composition and particle size fraction on bacterial abundances with a positive effect of ferrihydrite (oxic conditions). Our results indicate that the O_2_ status determined whether bacterial abundances were increased or decreased in HF samples. While under oxic condition bacterial abundances were higher in HF samples compared to bulk samples, under anoxic conditions they were lower maybe due to energy limitation that constrained the *Bacteria* in using mineral-associated OM (**Figures 4B,D**).

Taken together, the results of our microcosm incubation experiment revealed factors that possibly drive the abundance patterns at the Franz Josef chronosequence, i.e., O_2_ status and soil fraction for bacterial abundances, whereas archaeal abundances were hardly affected by the tested factors. High precipitation regularly creates water-saturated conditions with low oxygen availability in these soils. Consequently, the rather anoxic conditions and the increasing influence of the soil mineral phase with soil depth and soil age decreases bacterial abundances. Additionally, soil minerals such as Fe and Al oxides and other clay-sized minerals, e.g., vermiculite, could impair the substrate availability for soil microorganisms due to the formation of mineral-organic associations by adsorption and coprecipitation (Kaiser and Guggenberger, 2007; Heckman et al., 2013; Kleber et al., 2015; Dietel et al., 2017) and thereby creating or intensifying nutrient-limited conditions. However, substrates bound to mineral surfaces are partly still available for microbial utilization depending on the sorption–desorption properties of the bound substrates, but their decomposition is retarded (Mikutta et al., 2007; Dippold et al., 2014). Thus, while *Bacteria* might be inhibited, *Archaea* probably better cope with this mineral-induced nutrient and substrate limitation because of their slow growth rates, their rather oligotrophic lifestyle and their hypothesized better adaptation to chronic energetic stress resulting in increasing archaeal to bacterial abundance ratios in subsoils, especially at the oldest site of the Franz Josef chronosequence.

A decreasing fungal to bacterial ratio with soil depth along the Franz Josef chronosequence (**Figure 1C**) and higher ratios in LF samples compared to HF and bulk samples under oxic conditions in our incubation experiment (**Figure 4E**) point to a predominance of *Fungi* in habitats enriched in particulate OM (i.e., leaf and root litter) and with a high oxygen availability. These findings match with previous studies reporting relatively higher fungal abundance in soils with litter or soil macroaggregates (250–2000 μm, contain particulate OM) as compared to *Bacteria* (Baldrian et al., 2012; Smith et al., 2014). Noteworthy, under anoxic conditions we detected higher fungal to bacterial ratios for the C and CP treatment of the HF with almost similar SSU rRNA gene copy numbers for both taxa (**Figures 4B,C,E**). Accordingly, *Fungi* may survive adverse conditions as spores, but their abundance could increase even in anoxic habitats dominated by mineral-associated OM if there is an easy-degradable C source such as cellulose available. This observation corresponds to findings of Wild et al. (2014) for subsoil horizons that were amended with different ^13^C-labeled substrates with a significant, positive effect of glucose and amino acids on fungal abundance.

### Shift in Archaeal Community Composition with Ongoing Soil Development

The increasing archaeal to bacterial abundance ratio with soil age was accompanied by a clear compositional shift of the archaeal community (**Figure 2A**). At young to intermediate-aged soils the archaeal community composition was characterized by the occurrence of common soil groups as the *FSCG* and *SAGMCG-1* belonging to the phylum *Thaumarchaeota*. Surprisingly, at the oldest, P-limited site, the *Bathyarchaeota* were dominant with a proportion of up to 74%. Most of our sequences were affiliated with the pSL22 cluster (**Figure 3**), originally derived from a hot spring in Yellowstone National Park (Barns et al., 1996) and clustered to sequences derived from hydrothermal vents or other hot springs with no cultivated representatives. However, some of our bathyarchaeotal sequences were affiliated with sequences of the *MCG* subgroup 6 derived from marine and freshwater sediments, subsurface or wastewater sludge, thus, representing a rather widespread subgroup. So far *Bathyarchaeota* mostly comprising the *MCG* (Meng et al., 2014), that were reported to be one of the most abundant and at the same time one of the most active groups in nutrient-poor, low energy environments like marine subsurface sediments (Fry et al., 2008; Breuker et al., 2013; **Figure 3**). There are only a few studies, mainly about waterlogged soils, reporting the *MCG* constituting a mentionable proportion of the archaeal soil community (summarized by Kubo et al., 2012 in the Supplementary Information). In peatland soils of the Appalachian Mountains not only *Archaea* of the *Group 1.1c* and *SAGMCG-1* were detected but also *MCG* with a proportion of 37% (Hawkins et al., 2014). Another study about permafrost soils analyzed the archaeal community in different soil depths and reported that 26–73% of the sequences were affiliated to *MCG*-OTUs corresponding to our high proportions (Wei et al., 2014).

Unfortunately, little is known about the physiological capabilities of the *Bathyarchaeota* because there are no cultivated representatives so far. The *MCG* archaea are considered to be mainly heterotrophic anaerobes (Biddle et al., 2006; Lazar et al., 2016) and *MCG* cells of the marine subseafloor encoded extracellular protein-degrading enzymes such as aminopeptidases, thus, seem to be able to degrade persistent detrital matter comprised of cell wall components (Lloyd et al., 2013; Lazar et al., 2016). This assumption would be in line with an increase of aminopeptidase activities at the oldest site (Turner et al., 2014) suggesting that *Bathyarchaeota* at the oldest site may be adapted to the substrate and nutrient-poor conditions and are probably able to degrade proteins of dead microbial biomass that comprises an important internal carbon and nutrient pool at this soil development stage receiving only a small allochthonous OM input compared to the younger soil ages (Turner et al., 2013; calculated from Turner et al., 2014).

The archaeal community composition shift in soils of the Franz Josef chronosequence was related to soil age (**Figure 2C**) that is accompanied by a mineralogical and P gradient. Although the sample number for this pyrosequencing analysis was relatively low (*n* = 9), the age-related archaeal community composition shift matched with the T-RFLP results of our incubation experiment: The archaeal community composition was significantly affected by soil age and soil fraction, too (**Figure 6A**). Hemkemeyer et al. (2014) also reported an effect of different clay minerals on archaeal community composition in the particle size fraction <20 μm after 18 months of incubation. In contrast, the addition of C or P had no impact on the archaeal community composition (**Table 3**) consistent with findings of fertilization studies that showed that the archaeal community composition was not impacted by P addition (Leff et al., 2015). These results indicated that the age-related shift in archaeal community composition probably is caused by differences in soil mineralogical properties instead of changes in C or P contents.

### Bacterial Communities Remained Stable over Long-Term Soil Development

In contrast to the clear age-related compositional shift of the archaeal community, the bacterial community did not change considerably (**Figures 2B,D**). Our results revealed that neither on phylum nor on genus level a restructuring of the bacterial community composition took place in mineral soils over long-term soil development. Similarly, Jangid et al. (2013b) reported stable bacterial communities in topsoils of the Franz Josef chronosequence older than 5 kyr and this observation was also described for other long-term soil chronosequences such as the Mendocino chronosequence (uplifted marine terraces, time points T2 and T3), B horizons of a dune chronosequence located in Georgia (United States), and a dune chronosequence bordering on the Lake Michigan (Tarlera et al., 2008; Williams et al., 2013; Uroz et al., 2014). Thus, bacterial communities seem to change drastically during early succession, but then remain relatively stable despite of changing mineralogical composition and the nutrient gradients. This observation could be linked to the rapidly decreasing soil pH, from approximately seven to four, during early succession of glacier forelands (Tscherko et al., 2003; Williams et al., 2013; Turner et al., 2014); pH is considered to be one of the most important factors structuring bacterial community composition (Fierer et al., 2009; Dini-Andreote et al., 2014). This explanation is also in line with our observation that the pH is one of the best predictors for bacterial community composition at the Franz Josef chronosequence.

The results of the incubation experiment showed that the O_2_ status was the most important controlling parameter for bacterial community composition if the pH remained stable during long-term soil development (**Figure 6B** and **Table 3**). This observation corresponds to our findings at the Franz Josef chronosequence where bacterial community composition was rather influenced by soil depth forming a steep redox gradient in these regularly water-saturated soils (**Figure 2D**).

Besides O_2_ status, bacterial community composition differed significantly between soil fractions (**Table 3**) and T-RFs that were unique for either bulk or HF samples were detected (Supplementary Table S9). Blackwood and Paul (2003) also conducted a soil fractionation experiment and analyzed the bacterial community composition in four different fractions. They reported distinct communities for the HF and the LF concluding that these fractions provided distinct soil habitats due to different characteristics of the soil matrix and the OM within each fraction. They presumed that the selection for well adapted species may be greater in the HF compared to other fractions like the rhizosphere.

There are several studies reporting a significant effect of mineral composition such as different clay minerals or Fe oxides on bacterial communities in incubation experiments with artificial soils (Babin et al., 2013; Heckman et al., 2013; Vogel et al., 2014; Steinbach et al., 2015). On the contrary, we detected only a slight soil age effect for bacterial T-RFLP profiles analyzed with HhaI (**Table 3**). This difference may be related to the use of natural soils in our study which featured less pronounced differences in mineralogical compositions compared to artificial soils consisting of pure minerals used in other studies.

## Conclusion

Our study provides first insights in patterns of microbial communities in soil depth profiles along the long-term ecosystem development gradient of the Franz Josef chronosequence. The archaeal to bacterial abundance ratio increased not only with soil depth, but also with soil age with archaeal abundances being less impacted by the mineral-associated soil fraction and soil age than *Bacteria*. Moreover, pyrosequencing results revealed that *Archaea* and *Bacteria* show different patterns in relation to soil age: archaeal communities were clearly affected by soil age linked to changes in soil mineralogical properties, whereas bacterial community composition remained stable. Overall, our results indicate, that archaeal communities may relatively predominate subsoils, especially in nutrient-depleted old soils, by better coping with mineral-induced substrate limitations and due to changes in community composition to taxa (i.e., *Bathyarchaeota*) that are adapted to nutrient-poor, low energy habitats.

Further research is necessary to gain a better understanding of the processes and general patterns associated with long-term soil development and the particular role of microorganisms being of utmost importance for nutrient cycling. Special attention should be paid to the impact of mineral-organic associations on soil microbial communities and the underlying mechanisms controlling this interaction.

## Author Contributions

AS, RM, and GG designed this study. RM, SM-S, and ST performed soil sampling. ST and SM-S conducted laboratory work. ST analyzed the data with input from FS (statistics) and CL (archaeal taxonomy), and wrote the manuscript. All authors discussed the data and revised the manuscript.

## Conflict of Interest Statement

The authors declare that the research was conducted in the absence of any commercial or financial relationships that could be construed as a potential conflict of interest.
