# Supplementary material for: Microbial Community Dynamics in Soil Depth Profiles Over 120,000 Years of Ecosystem Development
Source: Front Microbiol. 2017 May 19;8:874. doi: 10.3389/fmicb.2017.00874 (PMC5437693; doi:10.3389/fmicb.2017.00874)
Supplement: Supplementary file 1 [file Table_1.pdf]

*Supplementary Material*

# Microbial Community Dynamics in Soil Depth Profiles over 120,000 Years of Ecosystem Development

**Stephanie Turner, Robert Mikutta, Sandra Meyer-Stüve, Georg Guggenberger, Frank Schaarschmidt, Cassandre Sara Lazar, Reiner Dohrmann, Axel Schippers***

*** Correspondence:** Axel Schippers, Axel.Schippers@bgr.de

## 1 Supplementary Tables and Figures

### 1.1 Supplementary Tables

**Table S1** Initial concentrations of organic carbon (OC) and total nitrogen (TN) per g soil fraction of samples used for the soil incubation experiment (Turner et al., 2017).

| Site age (kyr) | Fraction | OC (mg $g^{-1}$) | TN (mg $g^{-1}$) |
|---|---|---|---|
| 0.5 | Bulk | 144.2 | 7.9 |
| 5 | | 137.3 | 7.7 |
| 12 | | 77.7 | 3.6 |
| 120 | | 65.5 | 3.6 |
| 0.5 | HF | 126.1 | 6.7 |
| 5 | | 99.8 | 5.5 |
| 12 | | 63.7 | 3.0 |
| 120 | | 57.1 | 3.1 |
| 0.5 | LF | 403.5 | 14.0 |
| 5 | | 409.5 | 14.1 |
| 12 | | 387.9 | 11.9 |
| 120 | | 381.8 | 10.5 |

**Table S2** Physico-chemical and soil mineralogical properties of soil horizons along the Franz Josef chronosequence (Turner et al., 2014). Soil horizons used for the incubation experiment are highlighted in green. Hor. – horizon, OC – organic carbon, ON – organic nitrogen, TP – total phosphorus, $Fe_d$ – dithionite-extractable Fe phases representing poorly crystalline and crystalline Fe oxides as well as Fe-humus-complexes, $(Fe+Al)_o$ – oxalate-extractable Fe and Al phases representing poorly crystalline minerals and metal-humus-complexes, $Fe_{d-o}$ – difference between dithionite-extractable Fe and oxalate-extractable Fe representing crystalline Fe phases, $(Fe+Al)_p$ – Fe and Al from organic complexes.

| Site age (kyr) | Hor. | pH | OC (g kg$^{-1}$) | ON (g kg$^{-1}$) | TP (mg kg$^{-1}$) | $(Fe+Al)_o$ (g kg$^{-1}$) | $Fe_d$ (g kg$^{-1}$) | $(Fe+Al)_p$ (g kg$^{-1}$) | $Fe_{d-o}$ (g kg$^{-1}$) | Clay (%) | Silt (%) | Sand (%) |
|---|---|---|---|---|---|---|---|---|---|---|---|---|
| 0.06 | AO | 5.8 | 201.5 | 13.2 | 1103 | 4.2 | 3.3 | 4.3 | 0.4 | 11 | 31 | 58 |
| 0.06 | CA | 6.0 | 7.3 | 0.5 | 686 | 2.3 | 2.9 | 1.1 | 1.1 | 3 | 17 | 80 |
| 0.06 | C | 6.4 | 7.2 | 0.4 | 804 | 2.7 | 3.6 | 1.7 | 1.5 | 6 | 31 | 63 |
| 0.5 | OA | 4.5 | 241.7 | 10.2 | 785 | ND | ND | 11.6 | ND | ND | ND | ND |
| 0.5 | A | 4.7 | 158.4 | 6.9 | 583 | 7 | 3.6 | 7.7 | 0.0 | 17 | 55 | 28 |
| 0.5 | C | 5.6 | 3.6 | 0.2 | 744 | 1.7 | 1.3 | 1.5 | 0.1 | 5 | 46 | 49 |
| 1 | O | 3.9 | 327.6 | 13.4 | 544 | ND | ND | 2.9 | ND | ND | ND | ND |
| 1 | AE | 4.1 | 66.6 | 4.0 | 220 | 2.4 | 1.3 | 3.6 | 0.1 | 12 | 58 | 30 |
| 1 | E | 4.7 | 17.2 | 0.7 | 103 | 3.2 | 2.0 | 3.3 | 0.6 | 12 | 55 | 33 |
| 1 | B | 5.3 | 9.5 | 0.5 | 272 | 8.5 | 6.8 | 5.5 | 1.1 | 5 | 43 | 52 |
| 5 | O | 3.9 | 367.9 | 14.4 | 681 | ND | ND | 2.3 | ND | ND | ND | ND |
| 5 | AE | 4.3 | 47.3 | 2.8 | 195 | 2.7 | 1.9 | 1.4 | 0.4 | 13 | 56 | 30 |
| 5 | E | 4.9 | 16.6 | 0.6 | 100 | 4.2 | 3.2 | 1.8 | 0.6 | 13 | 58 | 29 |
| 5 | B | 5.1 | 8.1 | 0.4 | 231 | 8.5 | 7.2 | 2.2 | 1.1 | 6 | 51 | 43 |
| 12 | O | 3.8 | 448.1 | 12.8 | 499 | ND | ND | 1.2 | ND | ND | ND | ND |
| 12 | AE | 4.1 | 143.6 | 6.8 | 244 | 1.6 | 0.6 | 1.2 | 0.0 | 7 | 56 | 36 |
| 12 | EA | 4.5 | 30.5 | 0.9 | 76 | 1.4 | 0.7 | 0.9 | 0.4 | 5 | 52 | 43 |
| 12 | B | 5.0 | 36.4 | 1.3 | 328 | 13.4 | 8.2 | 4.7 | 2.6 | 9 | 34 | 57 |
| 12 | C | 5.5 | 4.7 | 0.2 | 664 | 6.6 | 0.8 | 0.6 | 0.3 | 8 | 28 | 64 |
| 60 | O | 4.0 | 220.3 | 6.2 | 296 | ND | ND | 1.6 | ND | ND | ND | ND |
| 60 | AE | 4.1 | 60.4 | 2.9 | 161 | 1.7 | 0.7 | 1 | 0.2 | 11 | 68 | 20 |
| 60 | EA | 4.7 | 24.0 | 1.0 | 92 | 5.9 | 3.8 | 3.1 | 0.5 | 14 | 67 | 19 |
| 60 | B | 5.1 | 13.3 | 0.7 | 137 | 13.9 | 9.4 | 3.9 | 1.8 | 16 | 59 | 25 |
| 60 | C | 5.5 | 2.2 | 0.1 | 591 | 2.6 | 1.2 | 0.9 | 0.5 | 7 | 43 | 50 |
| 120 | O | 4.4 | 190.4 | 5.4 | 136 | ND | ND | 0.6 | ND | ND | ND | ND |
| 120 | A | 4.2 | 72.1 | 4.0 | 78 | 0.5 | 0.2 | 0.5 | 0.0 | 10 | 77 | 13 |
| 120 | E | 4.9 | 7.7 | 0.4 | 16 | 2.9 | 2.3 | 1.7 | 1.4 | 12 | 74 | 13 |
| 120 | B | 5.2 | 12.5 | 0.4 | 77 | 7.8 | 24.7 | 4 | 22.0 | 20 | 51 | 29 |

ND = not determined

**Table S3** Results of the Tukey's post hoc test following the two-way ANOVA (soil age, horizon, their interaction and depth as a covariate) on microbial abundances along soil profiles of the Franz Josef chronosequence determined by qPCR (Figure 1). Letters indicate differences in abundances between different soil ages within each horizon (horizontal direction). Equal letters denote that the abundances not significantly differed from each other (Tukey test, $P < 0.05$).

| | Soil age (kyr) | | | | | | |
|---|---|---|---|---|---|---|---|
| | 0.06 | 0.5 | 1 | 5 | 12 | 60 | 120 |
| *Archaea* | | | | | | | |
| O | - | ab | a | a | a | ab | b |
| A | a | ab | ab | ab | b | a | a |
| E | - | - | a | a | a | a | a |
| B | - | - | a | a | a | a | a |
| C | a | a | - | - | b | a | - |
| *Bacteria* | | | | | | | |
| O | - | ab | a | a | a | ab | b |
| A | abc | abc | abc | ab | c | ac | b |
| E | - | - | a | ab | a | a | b |
| B | - | - | ab | ab | a | ab | b |
| C | a | ab | - | - | - | b | - |
| *Fungi* | | | | | | | |
| O | - | ab | a | a | a | ab | b |
| A | ab | ab | ab | a | ab | b | a |
| E | - | - | a | ab | a | a | b |
| B | - | - | a | a | a | a | a |
| C | a | ab | - | - | - | b | - |
| *Eukarya* | | | | | | | |
| O | - | a | a | a | a | a | a |
| A | ab | ab | ab | a | ab | b | ab |
| E | - | - | ab | ab | a | a | b |
| B | - | - | ab | ab | ab | a | b |
| C | a | ab | - | - | - | b | - |
| *Archaea:Bacteria* | | | | | | | |
| O | - | a | a | a | a | a | a |
| A | a | bc | bc | bc | b | ac | bc |
| E | - | - | ab | a | ab | a | b |
| B | - | - | a | a | a | a | b |
| C | a | b | - | - | - | b | - |
| *Fungi:Bacteria* | | | | | | | |
| O | - | a | a | a | a | a | a |
| A | a | a | a | a | a | a | a |
| E | - | - | a | a | a | a | a |
| B | - | - | ab | ab | ab | a | b |
| C | a | a | - | - | - | a | - |

**Table S4** Spearman rank order correlation coefficients for SSU rRNA gene copy numbers $g^{-1}$ dry weight soil and soil chemical and mineralogical properties of the soil chronosequence. Significant results at $P < 0.01$ are typed in bold.

| | $NO_3^-$ | $NH_4^+$ | TP | $(Fe+Al)_o$ | $Fe_d$ | $(Fe+Al)_p$ | $Fe_{d-o}$ | Clay | Silt | Sand |
|---|---|---|---|---|---|---|---|---|---|---|
| *Archaea* | 0.19 | **0.56** | -0.02 | -0.20 | -0.23 | 0.18 | **-0.33** | 0.14 | 0.23 | -0.20 |
| *Bacteria* | **0.36** | **0.69** | 0.18 | -0.17 | -0.27 | 0.08 | **-0.44** | 0.13 | 0.14 | -0.09 |
| *Fungi* | **0.40** | **0.78** | 0.08 | -0.16 | -0.28 | 0.00 | **-0.38** | **0.31** | **0.33** | **-0.31** |
| *Eukarya* | **0.42** | **0.76** | 0.06 | -0.06 | -0.14 | 0.04 | -0.30 | **0.30** | 0.24 | -0.22 |

**Table S5** Number of archaeal and bacterial observed OTUs, richness estimator, and diversity index of the 16S rRNA gene sequences of samples along the Franz Josef chronosequence.

| Sample | Archaea | | | Bacteria | | |
|---|---|---|---|---|---|---|
| | No. OTUs[1] | Chao1[2] | H[3] | No. OTUs[1] | Chao1[2] | H[3] |
| 0.5_CB | 323 | 745 (603-959) | 3.10 (3.04-3.17) | 635 | 2134 (1748-2653) | 5.54 (5.45-5.62) |
| 5_O | 239 | 565 (441-765) | 2.60 (2.54-2.67) | 922 | 4272 (3507-5264) | 6.28 (6.21-6.35) |
| 5_AE | 288 | 685 (541-911) | 3.12 (3.06-3.18) | 500 | 1359 (1116-1697) | 5.20 (5.12-5.29) |
| 5_E | 177 | 410 (308-590) | 2.56 (2.51-2.62) | 454 | 1276 (1030-1629) | 5.08 (5.00-5.16) |
| 5_B | 199 | 442 (343-609) | 2.62 (2.57-2.68) | 456 | 1429 (1134-1853) | 5.13 (5.06-5.10) |
| 12_EA | 343 | 1029 (799-1374) | 2.77 (2.70-2.84) | 498 | 1319 (1087-1642) | 5.18 (5.10-5.27) |
| 12_B | n.d. | n.d. | n.d. | 501 | 1815 (1424-2374) | 5.21 (5.13-5.29) |
| 120_E1 | 225 | 608 (459-852) | 2.26 (2.19-2.32) | 361 | 812 (662-1036) | 4.89 (4.81-4.96) |
| 120_E2 | 163 | 358 (275-503) | 1.69 (1.63-1.75) | 219 | 552 (421-767) | 3.68 (3.59-3.77) |
| 120_B | 176 | 419 (314-603) | 1.82 (1.75-1.89) | 267 | 850 (626-1214) | 4.18 (4.09-4.26) |

[1] Number of observed OTUs

[2] Chao1 richness estimator with higher and lower 95% confidence interval in parentheses.

[3] Shannon diversity index with higher and lower 95% confidence interval in parentheses.

n.d. no data

**Table S6** Effects of the different factors on microbial abundances in the soil microcosm incubation experiment (bulk and HF, without the LF) as revealed by five-way ANOVA with significant interactions. Frac – fraction.

| | $O_2$ | Fraction | Site age | P | C | Significant interactions |
|---|---|---|---|---|---|---|
| *Archaea* | * | n.s. | *** | n.s. | n.s. | $O_2$×Age*, Age×P*, $O_2$×Frac×Age***, $O_2$×Frac×P**, $O_2$×P×C***, Frac×Age×C*, Frac×Age×P** |
| *Bacteria*[1] | *** | *** | *** | *** | n.s. | $O_2$×Frac***, $O_2$×Age***, $O_2$×P***, Frac×Age***, Frac×P***, Age×C***, $O_2$×Frac×Age***, $O_2$×Age×P*, $O_2$×Age×C***, $O_2$×P×C***, Frac×Age×C*, Frac×P×C* |
| *Fungi*[1] | *** | n.s. | ** | *** | *** | $O_2$×Age***, $O_2$×C*, Frac×Age**, Frac×P**, Frac×C**, Age×C**, $O_2$×Frac×Age**, $O_2$×Frac×P*, $O_2$×Frac×C***, $O_2$×Age×P* |
| *Archaea*:*Bacteria*[1] | *** | ** | ** | n.s. | n.s. | $O_2$×Frac***, $O_2$×Age***, $O_2$×P*, Age×P**, $O_2$×Frac×Age**, $O_2$×Frac×P**, Frac×Age×P*, Frac×Age×C*, Frac×P×C* |
| *Fungi:Bacteria*[1] | *** | n.s. | *** | *** | *** | $O_2$×Frac***, $O_2$×P***, Frac×C**, Age×C***, P×C**, $O_2$×Frac×Age**, $O_2$×Frac×C***, $O_2$×Age×C** |

*** P < 0.001, ** 0.001 < P < 0.01; * 0.01 < P < 0.05; n.s. – not significant (P > 0.05).

[1] data not normal-distributed, see Table S7 for analysis of normal-distributed oxic and anoxic data subset.

**Table S7** Results of four-way ANOVA with significant interactions for the oxic and anoxic subsets of the soil incubation experiment (HF vs. bulk samples, without the LF). Frac – fraction.

| | Fraction | Site age | P | C | Significant interactions |
|---|---|---|---|---|---|
| *Bacteria* oxic | *** | *** | *** | ** | Frac×Age***, Age×C***, P×C***, Frac×Age×P* |
| *Bacteria* anoxic | *** | *** | n.s. | n.s. | Frac×P**, Age×P**, P×C**, Frac×P×C* |
| *Fungi* oxic | n.s. | *** | *** | *** | Frac×Age***, Frac×P*, Frac×C***, Age×P***, Age×C***, Frac×Age×C*, Frac×P×C***, Age×P×C* |
| *Fungi* anoxic | n.s. | *** | *** | *** | Frac×P**, Frac×C***, Age×C*; P×C* |
| *Archaea*:*Bacteria* oxic | n.s. | *** | * | n.s. | Frac×P*, Frac×Age×C* |
| *Archaea*:*Bacteria* anoxic | *** | *** | n.s. | n.s. | Frac×Age**, Frac×P*, Age×P*, Frac×Age×C* |
| *Fungi:Bacteria* oxic | *** | *** | ** | *** | Frac×Age***, Frac×C***, Age×P***, Age×C***, P×C***, Frac×Age×P**, Frac×Age×C***, Frac×P×C**, Age×P×C*** |
| *Fungi:Bacteria* anoxic | *** | * | *** | *** | Frac×Age*, Frac×C*** |

*** $P < 0.001$, ** $0.001 < P < 0.01$; * $0.01 < P < 0.05$; n.s. – not significant ($P > 0.05$).

**Table S8** Results of variance component estimation of factors (fraction, soil age, P and C addition and their four-way interactions) controlling microbial abundances and abundance ratios in the oxic and anoxic subsets (without the LF, controls and inoculum) for the soil microcosm incubation experiment.

| | % of total variation | | | | |
|---|---|---|---|---|---|
| **Oxic** | | | | | |
| | *Archaea* | *Bacteria* | *Fungi* | *Archaea:Bacteria* | *Fungi:Bacteria* |
| Fraction | 0 | 33 | 0 | 0 | 17 |
| Age | 6 | 5 | 9 | 31 | 1 |
| P | 0 | 14 | 12 | 2 | 0 |
| C | 0 | 0 | 50 | 0 | 32 |
| Fraction × Age | 10 | 13 | 10 | 0 | 0 |
| Fraction × P | 1 | 1 | 0 | 3 | 0 |
| Fraction × C | 0 | 0 | 2 | 0 | 8 |
| Age × P | 0 | 0 | 5 | 3 | 5 |
| Age × C | 0 | 15 | 2 | 0 | 19 |
| P × C | 0 | 9 | 0 | 0 | 5 |
| Fraction × Age × P | 14 | 0 | 0 | 5 | 0 |
| Fraction × Age × C | 3 | 0 | 2 | 5 | 1 |
| Fraction × P × C | 5 | 0 | 2 | 1 | 0 |
| Age × P × C | 0 | 0 | 2 | 0 | 2 |
| Fraction × Age × P × C | 0 | 3 | 0 | 0 | 5 |
| Residual | 62 | 8 | 4 | 49 | 3 |
| **Anoxic** | | | | | |
| | *Archaea* | *Bacteria* | *Fungi* | *Archaea:Bacteria* | *Fungi:Bacteria* |
| Fraction | 0 | 53 | 0 | 29 | 0 |
| Age | 0 | 35 | 5 | 3 | 0 |
| P | 0 | 0 | 16 | 0 | 18 |
| C | 0 | 0 | 3 | 0 | 1 |
| Fraction × Age | 10 | 0 | 2 | 10 | 6 |
| Fraction × P | 0 | 1 | 8 | 1 | 3 |
| Fraction × C | 0 | 0 | 21 | 0 | 35 |
| Age × P | 0 | 2 | 0 | 0 | 0 |
| Age × C | 0 | 0 | 6 | 0 | 2 |
| P × C | 7 | 0 | 5 | 0 | 0 |
| Fraction × Age × P | 5 | 0 | 0 | 4 | 0 |
| Fraction × Age × C | 0 | 1 | 1 | 0 | 0 |
| Fraction × P × C | 0 | 1 | 0 | 0 | 0 |
| Age × P × C | 0 | 0 | 0 | 0 | 0 |
| Fraction × Age × P × C | 20 | 0 | 0 | 17 | 1 |
| Residual | 58 | 7 | 34 | 36 | 34 |

**Table S9** Total number of T-RFs and number of T-RFs that were unique either in bulk, HF, or LF samples of the soil incubation experiment (without inoculum and control).

| | Total | Bulk | HF | LF |
|---|---|---|---|---|
| Archaea (HaeIII) | 31 | 3 | 3 | 1 |
| Archaea (RsaI) | 23 | 8 | 1 | 0 |
| Bacteria (HaeIII) | 52 | 5 | 6 | 0 |
| Bacteria (HhaI) | 44 | 7 | 4 | 1 |

## 1.2 Supplementary Figures

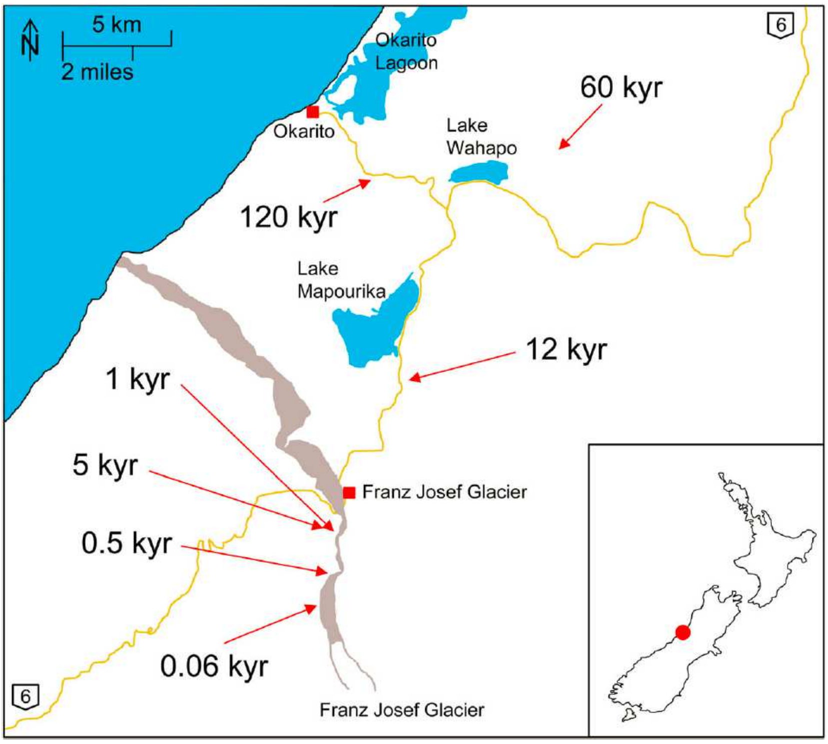


**Figure S1** Map of sampling sites along the 120 kyr old Franz Josef chronosequence modified after Dietel et al. (2017).

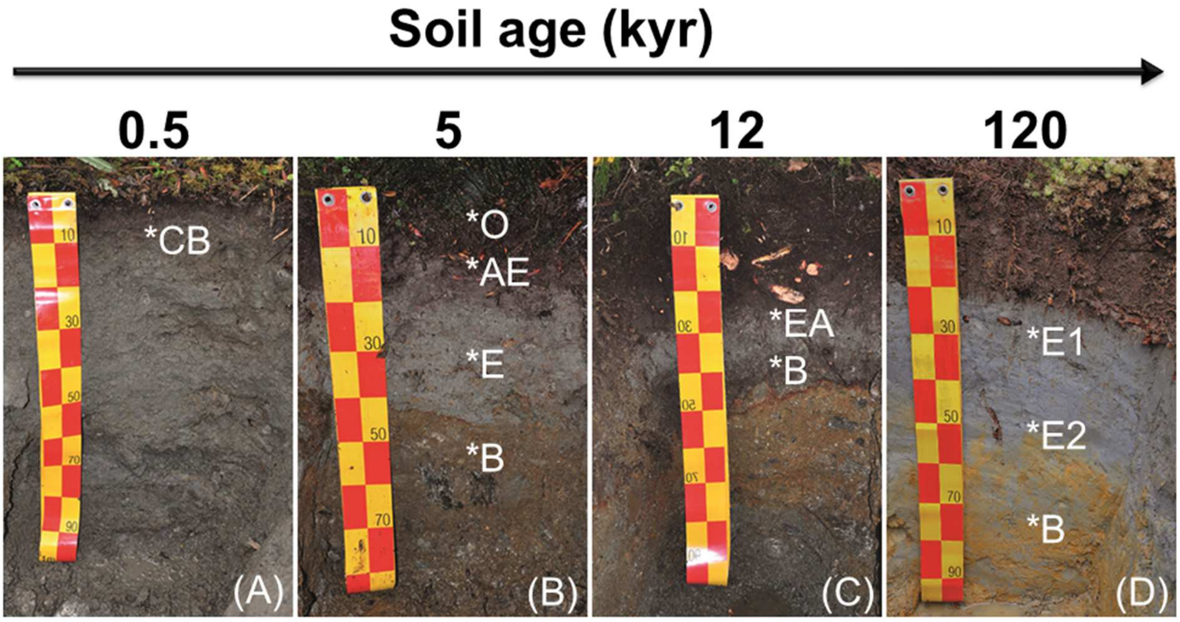


**Figure S2** Soil profiles at four sampling sites along the Franz Josef chronosequence of **(A)** 0.5, **(B)** 5, **(C)** 12, and **(D)** 120 kyr soil age. Asterisks mark horizons that were analyzed by pyrosequencing.

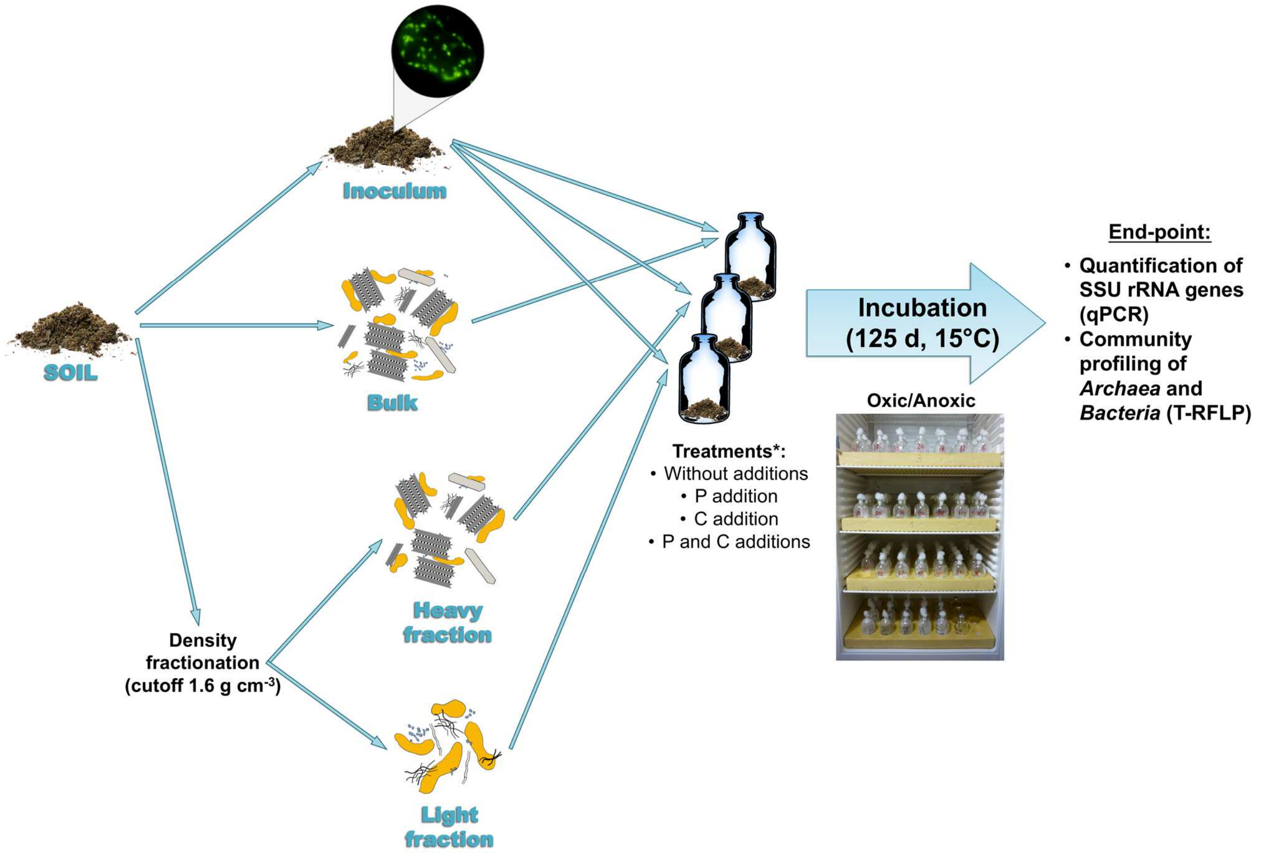


**Figure S3** Schematic overview of the microcosm incubation experiment setup modified after Turner et al. (2017). *The C and P treatments were not tested for the light fraction samples. Photographs courtesy of Christian Siebenbürgen and Norman Gentsch.

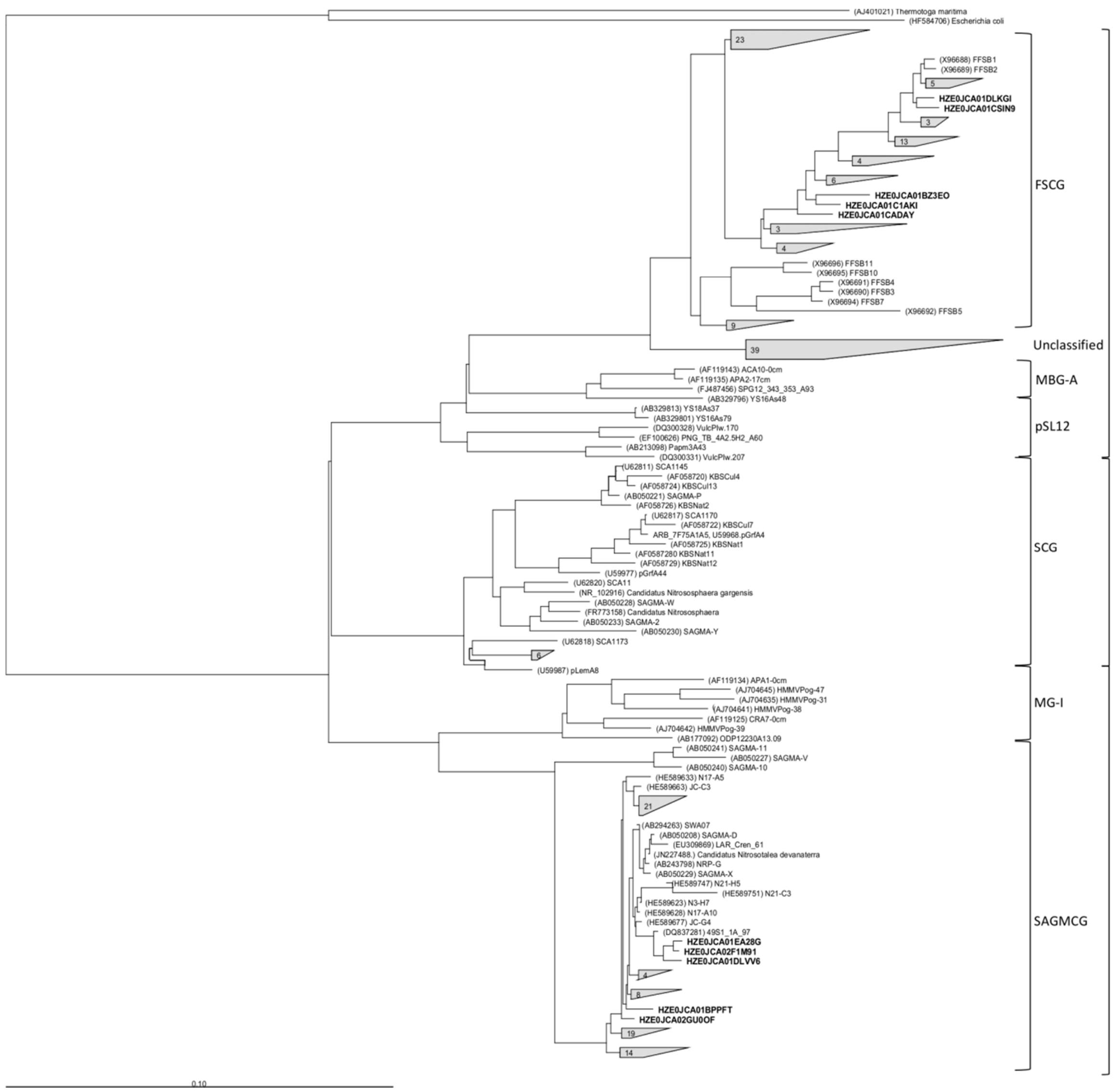


**Figure S4** Phylogenetic affiliation of *Thaumarchaeota* 16S rRNA gene sequences from soils along the Franz Josef chronosequence (in bold or grouped). The scale bar indicates 10% estimated phylogenetic divergence.

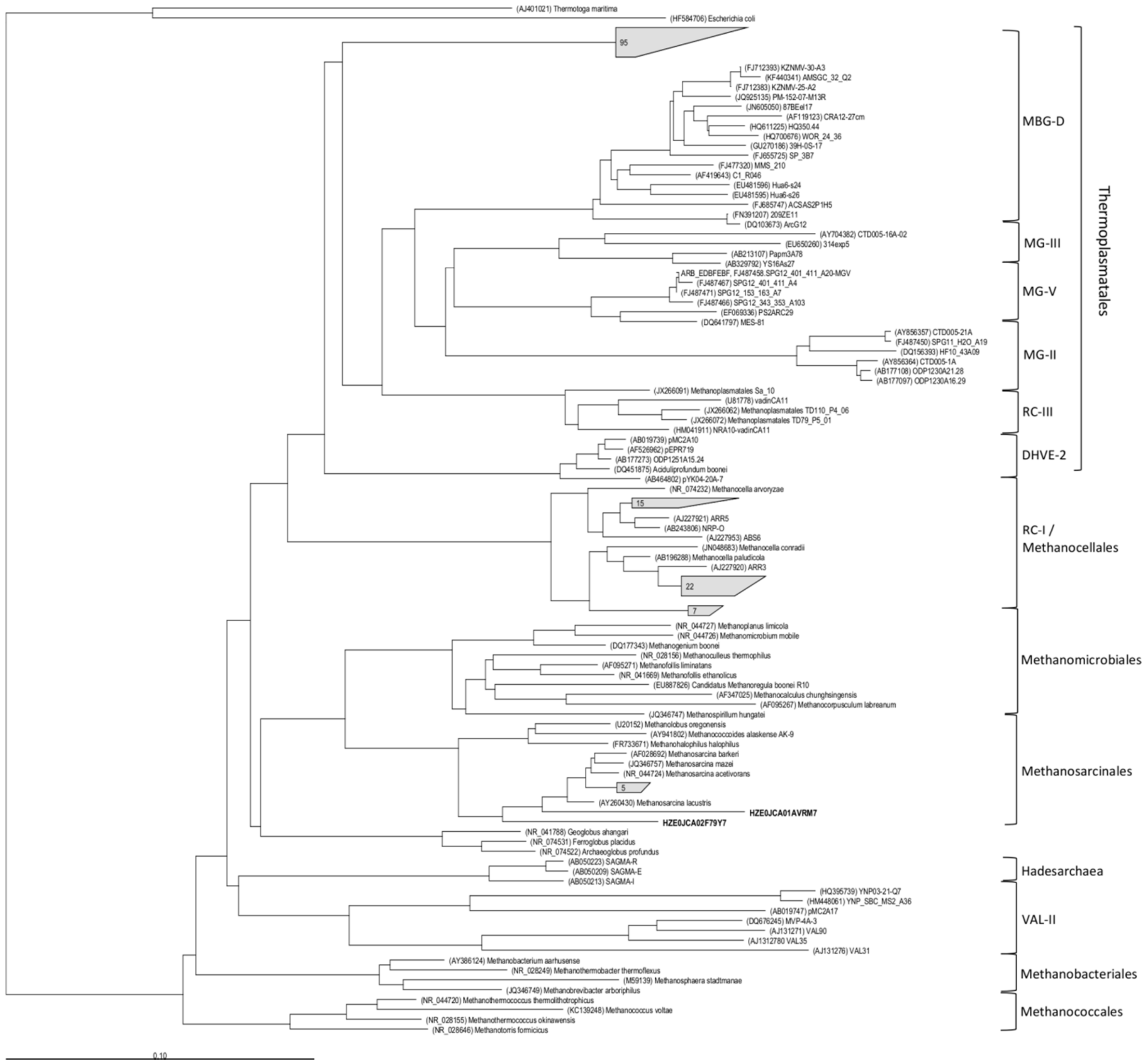


**Figure S5** Phylogenetic affiliation of *Euryarchaeota* 16S rRNA gene sequences from soils along the Franz Josef chronosequence (in bold or grouped). The scale bar indicates 10% estimated phylogenetic divergence.

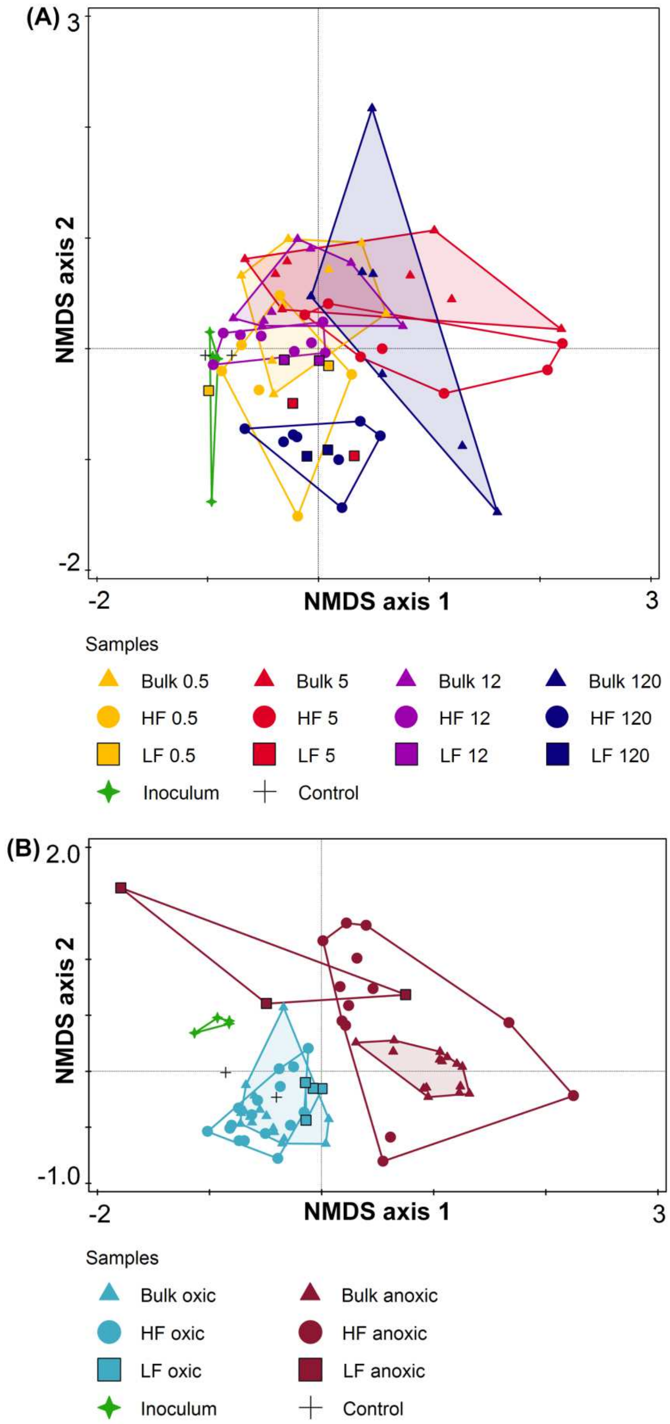


**Figure S6** Non-metric multidimensional scaling (NMDS) of **(A)** archaeal (stress value = 0.120) and **(B)** bacterial community composition (stress value = 0.100) for the soil microcosm incubation experiment based on relative abundance of T-RFs (after digestion of PCR products with RsaI or HhaI, respectively) using Bray-Curtis distance measure. Different symbols encode the different soil fractions (triangle – bulk soil; circle - heavy fraction, HF; and square – light fraction, LF). Different colors encode the different soil ages (0.5, 5, 12, and 120 kyr) for archaeal community analysis **(A)** and $O_2$ status (oxic and anoxic) for bacterial community analysis **(B)**.

**References**

Dietel, J., Dohrmann, R., Guggenberger, G., Meyer-Stüve, S., Turner, S., Schippers, A., et al. (2017). Complexity of clay mineral formation during 120,000 years of soil development along the Franz Josef chronosequence, New Zealand. *New Zeal. J. Geol. Geophys.* 60, 23–35. doi:10.1080/00288306.2016.1245668.

Turner, S., Meyer-Stüve, S., Schippers, A., Guggenberger, G., Schaarschmidt, F., Wild, B., et al. (2017). Microbial utilization of mineral-associated nitrogen in soils. *Soil Biol. Biochem.* 104, 185–196. doi:10.1016/j.soilbio.2016.10.010.

Turner, S., Schippers, A., Meyer-Stüve, S., Guggenberger, G., Gentsch, N., Dohrmann, R., et al. (2014). Mineralogical impact on long-term patterns of soil nitrogen and phosphorus enzyme activities. *Soil Biol. Biochem.* 68, 31–43. doi:10.1016/j.soilbio.2013.09.016.
